# High‐quality genome of a novel Thermosynechococcaceae species from Namibia and characterization of its protein expression patterns at elevated temperatures

**DOI:** 10.1002/mbo3.70000

**Published:** 2024-10-04

**Authors:** Nathanael D. Arnold, Michael Paper, Tobias Fuchs, Nadim Ahmad, Patrick Jung, Michael Lakatos, Katia Rodewald, Bernhard Rieger, Farah Qoura, Martha Kandawa‐Schulz, Norbert Mehlmer, Thomas B. Brück

**Affiliations:** ^1^ Department of Chemistry Werner Siemens‐Chair of Synthetic Biotechnology, TUM School of Natural Sciences Technical University of Munich Garching Germany; ^2^ Department of Integrative Biotechnology University of Applied Sciences Kaiserslautern Pirmasens Germany; ^3^ Department of Chemistry, WACKER‐Chair of Macromolecular Chemistry, TUM School of Natural Sciences Technical University of Munich Garching Germany; ^4^ Department of Chemistry and Biochemistry University of Namibia Windhoek Namibia

**Keywords:** cyanobacteria, genomics, proteomics, taxonomy, thermophilic, Thermosynechococcaceae

## Abstract

Thermophilic cyanobacteria thrive in extreme environments, making their thermoresistant enzymes valuable for industrial applications. Common habitats include hot springs, which act as evolutionary accelerators for speciation due to geographical isolation. The family Thermosynechococcaceae comprises thermophilic cyanobacteria known for their ability to thrive in high‐temperature environments. These bacteria are notable for their photosynthetic capabilities, significantly contributing to primary production in extreme habitats. Members of Thermosynechococcaceae exhibit unique adaptations that allow them to perform photosynthesis efficiently at elevated temperatures, making them subjects of interest for studies on microbial ecology, evolution, and potential biotechnological applications. In this study, the genome of a thermophilic cyanobacterium, isolated from a hot spring near Okahandja in Namibia, was sequenced using a PacBio Sequel IIe long‐read platform. Cultivations were performed at elevated temperatures of 40, 50, and 55°C, followed by proteome analyses based on the annotated genome. Phylogenetic investigations, informed by the 16S rRNA gene and aligned nucleotide identity (ANI), suggest that the novel cyanobacterium is a member of the family Thermosynechococcaceae. Furthermore, the new species was assigned to a separate branch, potentially representing a novel genus. Whole‐genome alignments supported this finding, revealing few conserved regions and multiple genetic rearrangement events. Additionally, 129 proteins were identified as differentially expressed in a temperature‐dependent manner. The results of this study broaden our understanding of cyanobacterial adaptation to extreme environments, providing a novel high‐quality genome of Thermosynechococcaceae cyanobacterium sp. Okahandja and several promising candidate proteins for expression and characterization studies.

## Graphical abstract

In this study, the genome of a new thermophilic cyanobacterium, Thermosynechococcaceae cyanobacterium sp. Okahandja, isolated from a hot spring near Okahandja in Namibia, was sequenced. Additionally, cultivations were conducted at elevated temperatures of 40, 50, and 55°C, followed by analyses of the respective adapted proteomes based on the annotated genome.

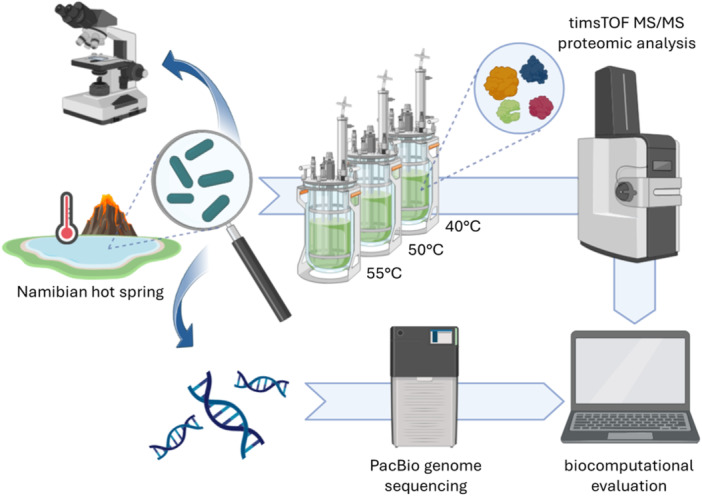

## 1 INTRODUCTION

Cyanobacteria are a diverse group of photosynthetic microorganisms that have significantly influenced the evolution of our planet through their impact on global carbon and nitrogen cycling, enabling life for oxygen‐dependent species (Knoll, 2008). Among them, thermophilic cyanobacteria have emerged as fascinating model organisms that thrive in extreme habitats characterized by high temperatures (Ward et al., 2012). They possess unique molecular and physiological adaptations, allowing them to adapt to environments where most other organisms cannot survive. Known mechanisms that confer thermophile cyanobacteria their ability to thrive in these adverse environments include cell membrane lipid composition alterations (homoviscous and homeophasic adaptation), higher GC content in rRNAs and tRNAs compared to mesophilic bacteria (Galtier & Lobry, 1997), changes in protein structure with more intramolecular salt bridges and fewer polar lipids per surface area (Thompson & Eisenberg, 1999), and potential production of more naturally heat‐resistant macromolecules as opposed to low MW molecules. Additionally, smaller genome sizes are a ubiquitous phenomenon for thermophilic organisms (Sabath et al., 2013; Van Noort et al., 2013), while functional simplification appears to be a common genomic adaptation mechanism in thermophilic prokaryotes (Burra et al., 2010).

Thermophilic microorganisms and their inherently thermostable enzymes are highly relevant for biotechnological applications since they allow for unsterile culture conditions without the threat of microbial contaminations, additionally requiring less energy‐intense cooling (Patel et al., 2019). In this context, the product spectrum of (thermophilic) cyanobacteria includes pigments (Graverholt & Eriksen, 2007), vitamins (Abed et al., 2009), poly‐β‐hydroxybutyrate (PHB) (Nishioka et al., 2001), bioactive compounds (Fish & Codd, 1994; Kumar et al., 2019; Singh et al., 2011), or lipids (Bywaters & Fritsen, 2015; Maslova et al., 2004). Further promising application fields comprise nutritional supplements (Watanabe et al., 1999), biofertilizers (Kumar et al., 2019), wastewater clearing (Aksu et al., 2009; Ertuǧrul et al., 2008; Sawayama et al., 1998), CO_2_ fixation (Hsueh et al., 2007; Leu et al., 2013), biofuel production (Karatay & Dönmez, 2011), or rare earth recycling (Paper et al., 2023). Despite their ubiquity and promising biotechnological applications, relatively little is known about the differential protein expression of cyanobacteria in response to elevated temperatures. While several studies and reviews (Tang et al., 2022; Wang et al., 2015) engaged with this scientific problem with the example of thermophilic bacteria (Chen et al., 2013; Gao et al., 2004; Pysz et al., 2004; Shih & Pan, 2011; Trauger et al., 2008; Wang et al., 2012), more research specifically aimed at cyanobacteria is required.

Cyanobacteria can be found in habitats with a wide range of environmental conditions (Callieri et al., 2019; Oren, 2015). Due to their geologically separated nature, hot springs are relevant island‐like ecosystems for speciation events, driven by local community dynamics (Ionescu et al., 2010). Members of the genus *Thermosynechcococcus* have been documented in hot springs with temperatures ranging from 44°C to 98°C (Saxena et al., 2017; Stolyar et al., 2014; Tang et al., 2018; Ward et al., 2017). Given their limited evolutionary distance, they give insights into how rapid environmental adaption can be achieved.

The investigated cyanobacterial strain of this study was isolated from a hot spring near the city of Okahandja in Namibia. We aimed to gain a comprehensive system biology‐driven understanding of the temperature adaptation of this newly isolated strain. Therefore, a multidisciplinary approach was deployed, combining PacBio‐guided long‐read genome sequencing and high‐resolution nLC‐Q‐TOF mass spectroscopy‐driven proteome analyses to unravel the distinct genetic features and metabolic adaptations that allow this thermophilic cyanobacterium to thrive in environments characterized by elevated temperature regimes. Through comparison of our high‐quality genomic data and the intracellular proteomes derived from cultures grown at different temperature profiles, we identified key genes and metabolic pathways involved in the adaptation to extreme temperatures and discussed the results in the context of comparable studies. In addition, sequence alignment with the closely related strain *Parathermosynechococcus lividus* PCC 6715 revealed extensive gene flux and rearrangements.

Maximum likelihood analysis based on 16S rRNA gene sequences and the concatenated phylogenetic tree indicated allopatric speciation, which was reinforced by ortholog‐based phylogenetics of Thermosynechococcaceae. Accordingly, the novel specimen was placed close to the branch of the recently defined genus of *Parathermosynechococcus*, which incorporates the Yellowstone National Park‐originating *P. lividus* PCC 6715 (Tang et al., 2024). Based on these combined findings, we suggest the name Thermosynechococcaceae cyanobacterium sp. Okahandja for the novel thermophilic cyanobacterium presented in this study.

## 2 MATERIALS AND METHODS

All chemicals were retrieved from Sigma‐Aldrich, while VWR supplied general consumables. All necessary buffers and enzymes for next‐generation genome sequencing were shipped from Pacific Biosciences. High‐molecular‐weight DNA was extracted using the Quick‐DNA™ HMW MagBead Kit from Zymo Research. HMW gDNA shearing was conducted using g‐TUBEs (Covaris). Polymerase chain reactions were done using the DreamTaq Polymerase and corresponding buffer (Thermo Fisher Scientific). For agarose gel DNA extraction, the Monarch DNA Gel Extraction Kit (New England BioLabs GmbH) was utilized according to the manufacturer's protocol.

### 2.1 Cultivation

The cyanobacteria were isolated from a hot spring near Okahandja in Namibia (Callieri et al., 2019) and cultivated in an Infors Lab5 stirred photobioreactor system (Infors HT) in BG11 medium according to Stanier et al. (1971) at temperatures of 40°C, 50°C, and 55°C. Stirring speed was set to 150 rpm and the illumination intensity was 200 µmol m^−2^ s^−1^ PPFD. During cultivation, the culture was aerated with 1.016 Nl/min through a sparger. By adjusting the in‐gas CO_2_ portion, the pH was maintained at 8.0. For proteome analysis, the biomass was harvested during the exponential growth phase after 7 days.

### 2.2 Microscopy

Microscopic images were taken using a confocal laser scanning microscope LSM 980 (Carl Zeiss) equipped with an Axiocam 508 color imaging system. Secondary electron (SE) and transmission electron images were taken using a JSM‐7500F Field Emission Electron Microscope (Jeol Ltd.) equipped with a low‐energy ionization detector (LEI) and a transmission electron detector in scanning mode (STEM). The SE images were taken with an accelerating voltage of 1 kV and the STEM images were taken with 30 kV. Before sample preparation, the culture was washed with demineralized water to remove salts.

### 2.3 Molecular biology analysis

#### 2.3.1 Species identification

##### 16S ribosomal RNA gene‐inferred phylogenetic analysis

The full‐length 16S ribosomal RNA gene sequence was extracted from the whole‐genome nucleotide sequence using Barrnap (Seemann, 2013), as implemented on the public Galaxy.eu server of the Galaxy web platform (Afgan et al., 2016). Using the BLAST tool of the National Center for Biotechnology Information (NCBI) GenBank, the 16S rRNA gene sequence was compared to already submitted sequences of cyanobacterial strains from public culture collections in terms of authenticity. The assembled 16S rRNA gene sequence and related sequences of cyanobacterial strains cited from GenBank were used for phylogenetic analysis, including *Gloeobacter* spp. as an outgroup. For the 16S rRNA gene alignment, the Muscle algorithm in Mega X (Kumar et al., 2018) was applied. The phylogenetic tree was calculated in Mega X using the Maximum Likelihood method (ML) and the General Time Reversible model (Nei & Kumar, 2000) with Gamma‐distributed rates and Invariant sites (GTR + G + I model) and a bootstrap value of 1000. For the Bayesian phylogenetic analyses, two runs of eight Markov chains were executed for one million generations with default parameters with Mr. Bayes 3.2.1 (Ronquist & Huelsenbeck, 2003) as described in Jung et al., 2024 (Jung et al., 2024).

##### Orthology‐inferred phylogenetic analysis

Using the public Galaxy.eu server of the Galaxy web platform (Afgan et al., 2016), OrthoFinder 2.5.4 (Emms & Kelly, 2015) was utilized to identify orthologs among the genomes of Thermosynechococcaceae as conducted by Jahodářová et al. (2022). Gene sequence searching was carried out using DIAMOND 2.0.15 (Buchfink et al., 2014) to find orthogroups (groups of genes descended from a single gene in the last common ancestor of a group of species). Unrooted gene trees were then inferred using DendroBLAST (Kelly & Maini, 2013). OrthoFinder utilizes STAG (Emms & Kelly, 2018) on default to infer unrooted species trees from all genes from the set of unrooted gene trees. These unrooted species trees are then rooted utilizing STRIDE (Emms & Kelly, 2017), from which OrthoFinder infers orthologs in a final step. See Emms and Kelly (2015) for details on the orthogroup inference stage and Emms and Kelly, (2019) for details on the second stage from orthogroups to gene trees, the rooted species tree, orthologs, gene duplication events, and so forth. In total, OrthoFinder assigned 85,177 genes (95.9% of the total) to 7952 orthogroups. Fifty percent of all genes were in orthogroups with 22 or more genes (G50 of all genes was 22) and were contained in the largest 1508 orthogroups (O50 was 1508). There were 451 orthogroups present within all compared Thermosynechococcaceae species' genomes, and 271 of these consisted entirely of single‐copy genes (exactly one from each compared species). These 271 single‐copy orthologs were utilized to infer the outgroup‐free, phylogenetic species tree depicted in Figure 4. iTol (Letunic & Bork, 2024) was deployed to visualize the Newick formatted phylogenetic species tree acquired from OrthoFinder.

#### 2.3.2 Next‐generation genome sequencing

##### HMW genomic DNA extraction and DNA library preparation

About 50 mL of cyanobacterial culture was collected during the exponential growth phase and centrifuged at 10.000 rcf for 5 min. The biomass was used for the extraction of high‐molecular‐weight genomic DNA with the Quick‐DNA HMW MagBead Kit (Zymo Research) following the manufacturer's instructions. Light absorption ratios at 260/280 nm wavelength were measured using a photometer (Nano Photometer NP80; IMPLEN) and a Qubit 4 fluorometer with the Qubit 1X dsDNA HS Assay‐Kit (Thermo Fisher Scientific) to evaluate the purity and concentration of the obtained genomic DNA. Fragment sizes of the gDNA were analyzed using a FemtoPulse capillary electrophoresis instrument (Agilent Technologies). Samples that passed the quality control were then sheared using g‐TUBEs (Covaris) with 1700 g in a tabletop centrifuge, resulting in DNA fragments of approximately 12 kb in size, as verified by FemtoPulse again. Subsequently, a HiFi library was then generated by following the instructions in the manual of the SMRTbell prep kit 3.0 (Pacific Biosciences), which involves attaching barcoded adapters to the gDNA fragments. The libraries were stored at −20°C until the day of sequencing, when the Sequel II Binding Kit 3.2 (Pacific Biosciences) was used to bind primers and polymerase to the samples, following the manufacturer's recommendations.

##### Sequencing

A single SMRT cell (lot number 418096) was used to perform whole‐genome sequencing on a Sequel IIe platform (Pacific Biosciences) utilizing the following parameters: 2 h of pre‐extension, 2 h of adaptive loading (with a target of p1 + p2 = 0.95) to obtain a final on‐plate concentration of 85 pM, and a movie window of 30 h for signal detection (Saxena et al., 2017).

##### Assembly and annotation

Obtained FASTQ raw read files were assembled utilizing the Canu assembler 2.0 (Tang et al., 2018) with a user‐provided estimated genome size of 2.7 Mbp (*genomeSize* = *2.7 mb*) and the *‐pacbio* parameter. The corresponding assembly report file can be found in the supplementary data at https://zenodo.org/doi/10.5281/zenodo.10007199. Gene prediction and annotation were performed using NCBI's Prokaryotic Genome Annotation Pipeline (PGAP) (Seemann, 2013; Stanier et al., 1971; Stolyar et al., 2014; Tang et al., 2024), comprising GeneMarkS‐2+ for gene prediction (Afgan et al., 2016) and TIGRFAMs for functional identification of proteins (Jung et al., 2024; Kumar et al., 2018; Nei & Kumar, 2000; Ronquist & Huelsenbeck, 2003). Secondary annotation was performed using the browser‐based tool RAST (Rapid Annotation using Subsystem Technology) (Buchfink et al., 2014; Emms & Kelly, 2015; Jahodářová et al., 2022) for detailed identification of genetic CRISPR elements in depth. Complementary functional annotation and phylogenetic classification of the genome‐encoded enzymes according to the Clusters of Orthologous Groups of proteins (COGs) were performed using eggNOG‐mapper (Emms & Kelly, 2018; Kelly & Maini, 2013). The full‐length ribosomal 16S rRNA gene sequence of the investigated strain, as extracted from the genome with barrnap (Seemann, 2013), showed highest similarities with an uncultivated cyanobacterium from the Sagole hotspring (Jarett et al., 2014), South Africa, with an identity of 99.6% and *P. lividus* PCC 6715 with an identity of 98.78% and according to a blastn suite search, utilizing the rRNA/ITS databases as reference (Zhang et al., 2000).

##### Quality control and in silico analyses

Deploying the public Galaxy.eu server of the Galaxy web platform (Emms & Kelly, 2017), the genome assembly quality was assessed regarding completeness and contamination with checkM (Emms & Kelly, 2019). In that context, CheckM was run through the browser‐based tool Protologger, which is also part of the Galaxy network (Letunic & Bork, 2024). Additionally, the genome completeness was analyzed based on orthologous genes with BUSCO 5.4.6 (Ritz et al., 2023), utilizing the cyanobacteria_odb10 lineage. Whole‐genome alignments were performed using the progressiveMauve (Koren et al., 2017) plugin within the Geneious Prime 2022.0.1 software. The circular plot was created using CIRCOS in a Linux Ubuntu environment (Li et al., 2021). Biosynthetic gene clusters were identified utilizing antiSMASH 7.0 (Tatusova et al., 2016).

### 2.4 Proteomics

#### 2.4.1 Sample preparation

For the proteome analysis, 50 mL of cyanobacterial culture was harvested and centrifuged at 5000*g* for 10 min. Afterward, the biomass pellet was frozen with liquid nitrogen and stored at −80°C. Proteome analysis was performed in triplicates following a previously described protocol (Haft et al., 2018). For sample preparation, the pellets were resuspended in 600 µL in aqueous solution with 2% SDS and 10 mM β‐mercaptoethanol. Next, the samples were incubated at 65°C for 5 min and centrifuged at 14,000 rcf for 10 min. The pellet was resuspended in 600 µL of methanol and vortexed. Then, 150 µL of chloroform was added and the sample was vortexed again. At last, 450 µL of dH_2_O was added. After vortexing, the samples were centrifuged at 14,000 rcf for 1 min. This step resulted in three separate phases. The middle phase, containing the proteins, was carefully isolated and mixed with 400 µL of methanol. Afterward, the samples were centrifuged at 14,000*g* for 2 min. After removing the supernatant, this step was repeated twice. Then, the samples were mixed with 400 µL of acetone, centrifuged at 14,000 rcf for 2 min, and the supernatant was discarded. This step was also repeated twice. The pellet was dried at room temperature for 25 min. Then, the pellet was resuspended in an aqueous solution with 8 M urea and 10 mM β‐mercaptoethanol and stored at −20°C.

Proteins were run 1 cm into a 10% Criterion™ Tris–HCl Protein Gel (Bio‐Rad Laboratories Inc.) and stained with Coomassie Brilliant Blue (SERVA Electrophoresis GmbH). Then, protein bands were excised from the gel and subsequently used for peptide isolation. The excised gel pieces were cut into small pieces (<1 mm^3^) and washed with acetonitrile until the Coomassie Brilliant Blue was fully removed. Afterward, the gel pieces were dried under vacuum for 15 min (GeneVac Evaporator; GeneVac HiTechTrader).

The dried samples were resuspended in 100 µL 10 mM Dithiothreitol solution and incubated for 45 min at 56°C in a shaker at 550 rpm. Then, the samples were washed with acetonitrile and incubated in 100 µL 55 mM iodoacetamide and 50 mM ammonium bicarbonate for 30 min in the dark in a shaker at 550 rpm. Afterward, the samples were washed twice with acetonitrile and dried under vacuum for 15 min (GeneVac Evaporator; GeneVac HiTechTrader). Then, samples were resuspended in 100 µL of digest solution containing 1 µL trypsin solution in 100 µL 25 mM ammonium bicarbonate buffer. The enzymatic digestion was performed overnight at 37°C in a thermal shaker at 300 rpm. Afterward, the peptides were extracted by adding 25 mM ammonium bicarbonate and a subsequent sonification for 15 min. After the addition of 100 µL acetonitrile and incubation for 15 min, 100 µL 5% formic acid was added, followed by a second sonification for 15 min. Then, 100 µL acetonitrile was added and the supernatant was transferred to a new microreaction tube. This step was repeated, and the samples were dried under vacuum (GeneVac Evaporator; GeneVac HiTechTrader). The dried peptide samples were mixed with 20 µL 1% formic acid, sonicated for 10 min, and filtered through a 10 kDa spin filter. Subsequently, the samples were subjected to LC‐MS/MS analysis.

#### 2.4.2 LC‐MS/MS analysis

Peptide analysis was done using an LC‐MS/MS analysis with a timsTOF Pro mass spectrometer equipped with a NanoElute LC System (Bruker Daltonik GmbH) on an Aurora column (250 × 0.075 mm, 1.6 µm; IonOpticks). The mobile phase comprised a 0.1% (v/v) water–formic acid mixture (A) and a 0.1% (v/v) acetonitrile–formic acid mixture (B), which was added as a binary gradient at a flow rate of 0.4 μL/min. The gradient concentration started at 2% (v/v) B and was increased linearly to 17% B after 36 min. After another 18 min, the percentage of C was increased to 25% (v/v) and then increased linearly to 37% B after 6 min. After 70 min, the concentration of B was adjusted to the final value of 95% (v/v). The oven temperature during the measurements was 50°C.

The timsTOF Pro mass spectrometer (TIMS) was used in PASEF mode with the following settings: mass range: 100–1700 mass: charge [*m*/*z*] ratio; ion mobility ramp, 0.6–1.6 V s/cm^2^; 10 MS/MS scans per ion mobility ramp (total cycle time 1.16 s); charge range, 0–5; active exclusion for 0.4 min; a target intensity of 20,000 counts; and an intensity threshold of 1000 counts. The collision energy was ramped stepwise, appropriate to the ion‐mobility ramp, from 20 to 59 eV. The electrospray ionization source parameters were 1600 V for the capillary voltage and 3 L/min N_2_ (as dry gas) at a dry gas temperature of 180°C. The measurements were performed in a positive ion mode. Mass calibration was performed using the sodium formate cluster, and the TIMS was calibrated using Hexais (2,2‐difluoroethoxy) phosphazene, Hexakis (2,2,3,3‐tetrafluoropropoxy) phosphazene, and Chip cube high mass references (m/z ratios of 622, 922, and 1222, respectively).

#### 2.4.3 Bioinformatic analyses

The MS/MS tandem spectrometry data of tryptic‐digested peptides were evaluated using the PEAKS studio software (v.10.6, build 20201221). The annotated genome of Thermosynechococcaceae cyanobacterium sp. Okahandja (this study) (BioSample accession SAMN37714564) served as a reference for protein identification (Ciufo et al., 2018). The database search parameters included a precursor mass of 25 ppm, employing monoisotopic mass, and a fragment ion error tolerance of 0.05 Da. Trypsin was used as the digestion enzyme, allowing a maximum of two missed cleavages per peptide. Up to three variable PTMs were allowed per peptide, and FDR estimation was performed using decoy fusion. For protein identification, a 1.0% FDR threshold was applied, requiring at least one unique peptide per protein. The included PEAKSQ software was used to differentially quantify the three temperature sample groups, consisting of technical triplicates, each. A mass error tolerance of 20.0 ppm, an ion mobility tolerance of 0.05 Da, and a retention time shift tolerance of 6 min (auto detect) were employed. Fold change and significance thresholds were set to 2 and 15, respectively, utilizing the analysis of variance significance method. All proteins were exported for manual evaluation and plot creation. RStudio (Lomsadze et al., 2018) with the ggplot2 package (Haft, 2001) and Inkscape 1.2.2 were utilized to create all heatmaps, bar charts, and plots unless stated otherwise.

## 3 RESULTS AND DISCUSSION

### 3.1 Morphology

**Figure 1:**
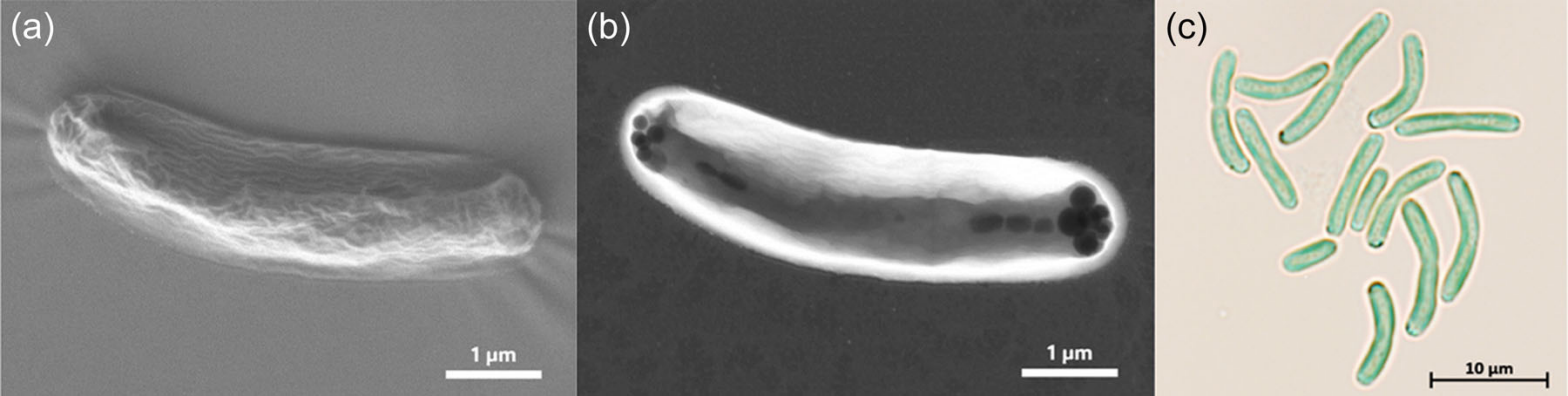
Microscopic images of Thermosynechococcaceae cyanobacterium sp. Okahandja using an electron microscope. (a) Secondary electron image using a low‐energy ionization (LEI) detector, (b) STEM image; (c) brightfield image using a light microscope.

**Figure 2:**
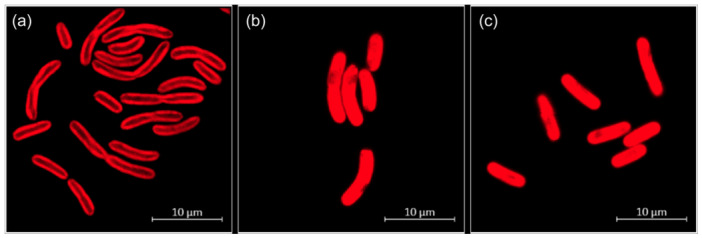
Fluorescence microscopic images of Thermosynechococcaceae cyanobacterium sp. Okahandja, grown at (a) 40°C, (b) 50°C, and (c) 55°C. The autofluorescence of chlorophyll results in red emissions; magnification ×1000.

The cyanobacteria cells had a cylindrical, usually feebly curved form with a diameter of 1–2 µm and a length of 4–9 µm (Figure 1c). Dividing cells were frequently connected as linear pairs. Dark granula at the poles of the cells (Figure 1b) might represent cell deposits for energy storage, such as glucans or polyhydroxybutyrate (PHB). The distribution of thylakoids within the cells differed depending on the temperature (Figure 2). At a cultivation temperature of 40°C, the thylakoids were predominantly located in the areas near the cell membrane. At 50°C and 55°C however, they appeared to be evenly distributed. Nevertheless, temperature differences did not affect the overall cell shape and size.

### 3.2 Whole‐genome sequencing

Whole‐genome sequencing with the PacBio Sequel IIe instrument yielded 859,882 >Q20 reads comprising over 6.4 billion base pairs, with a median read quality of Q36 and a mean >Q20 read length of 10,893 bp. The Canu v2.0 assembler (Tang et al., 2018) was fed with the raw read FASTQ files and recognized 49,509 reads with 540 million bases, equal to a 200‐fold genome coverage. After trimming, roughly 108 million bases from 6242 reads were further processed, corresponding to a 40‐fold genome coverage. One circular contig with a size of 2.8 Mbp could be constructed, similar in size to the *P. lividus* PCC 6715 reference genome size of 2.65 Mbp on NCBI (RefSeq NZ_CP018092.1).

**Figure 3:**
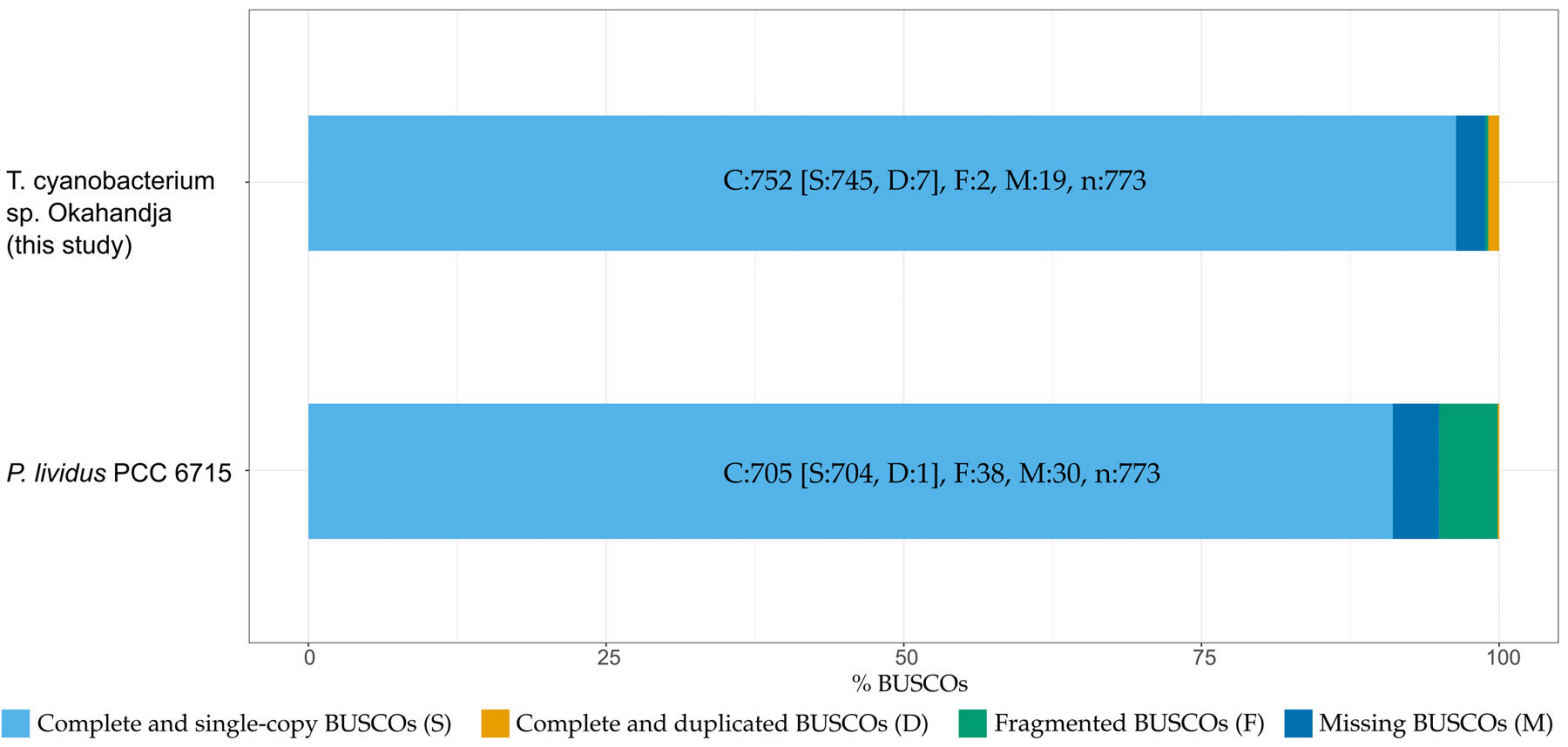
BUSCO 5.4.6 results for Thermosynechococcaceae cyanobacterium sp. Okahandja (this study) and the reference genome of *P. lividus* PCC 6715. Genome completeness assessment based on near‐universal single‐copy ortholog genes from the cyanobacteria lineage data set odb_10, comprising 141 cyanobacterial genomes. According to BUSCO 5.4.6 (Manni et al., 2021), the genome of this study exhibits less fragmented and overall more orthologous genes compared to the reference genome of *P. lividus* PCC 6715 (RefSeq NZ_CP018092.1). Both genomes were obtained from PacBio long‐read sequencing with genome assemblies resulting in a single, circular chromosome, respectively.

Genome quality was assessed with the tools checkM, according to which the genome completeness amounts to 99.76% with a contamination of 1.06% (Emms & Kelly, 2019), in addition to BUSCO v.5.4.6 which operates based on near‐universal single‐copy orthologs (Saxena et al., 2017). Comparing the gene set of the investigated Thermosynechococcaceae cyanobacterium sp. Okahandja genome to the cyanobacteria lineage data set (cyanobacteria_odb10) which consists of 141 genomes, a genome completeness of 97.3% was predicted (Figure 3). Interestingly, the reference genome of *P. lividus* PCC 6715 is estimated to possess merely 91.2% of all orthologous cyanobacterial genes, with a higher proportion of fragmented BUSCOs.

### 3.3 Phylogenetic analysis

#### 3.3.1 16S rRNA gene sequence‐inferred phylogeny

**Figure 4:**
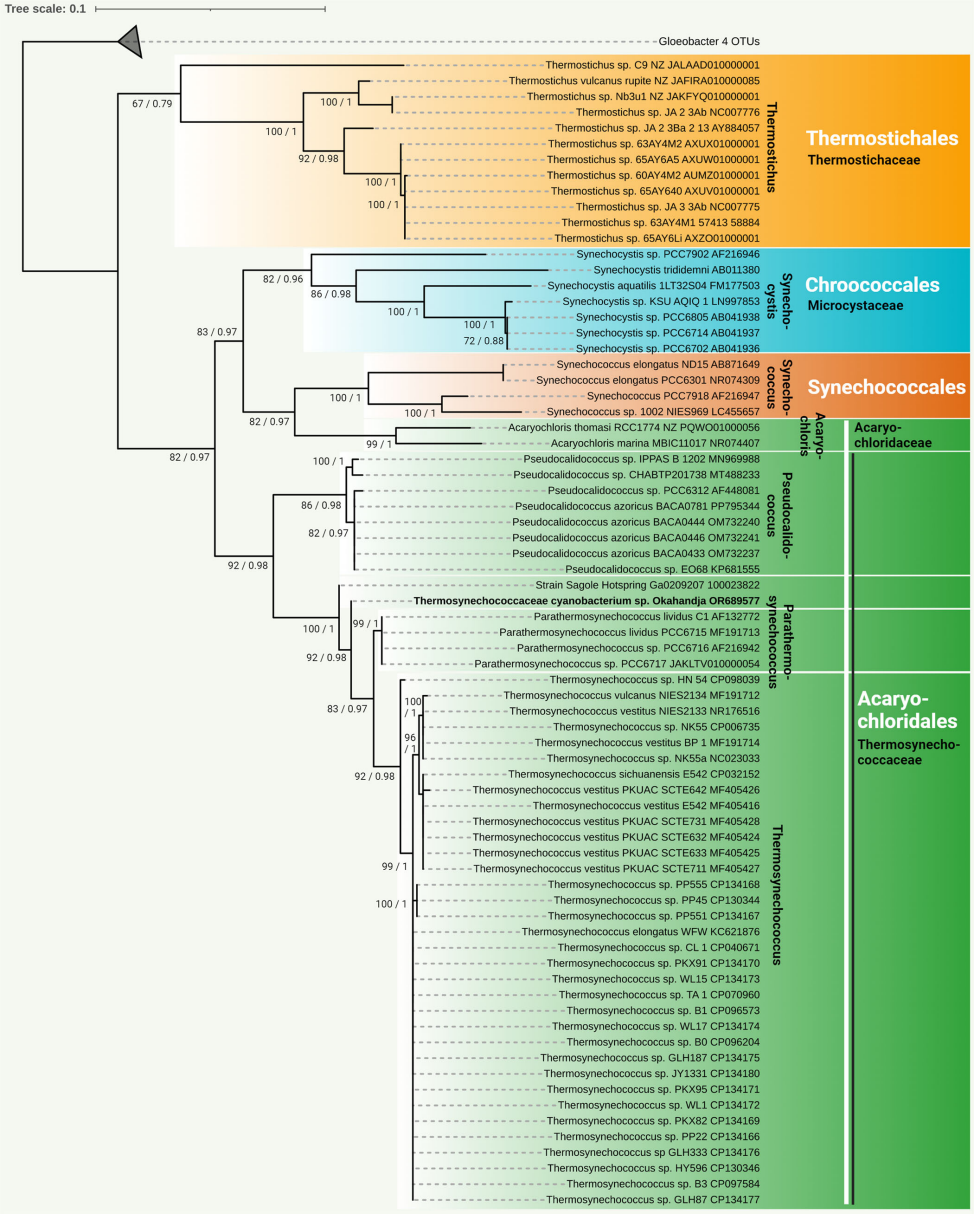
The evolutionary history was inferred by using the Maximum Likelihood method and General Time Reversible model (Nei & Kumar, 2000). Maximum Likelihood bootstrap values are given at the nodes together with Bayesian posterior probabilities. The tree with the highest log likelihood (−8988.74) is shown. The percentage of trees in which the associated taxa clustered together is shown next to the branches. Initial tree(s) for the heuristic search were obtained automatically by applying Neighbor‐Join and BioNJ algorithms to a matrix of pairwise distances estimated using the Maximum Composite Likelihood (MCL) approach and then selecting the topology with superior log likelihood value. A discrete Gamma distribution was used to model evolutionary rate differences among sites (5 categories [+G, parameter = 0.2925}). The rate variation model allowed for some sites to be evolutionarily invariable ([+I], 39.30% sites). The tree is drawn to scale, with branch lengths measured in the number of substitutions per site. This analysis involved 48 nucleotide sequences. There were a total of 2071 positions in the final data set. Evolutionary analyses were conducted in MEGA X (Kumar et al., 2018).

The complete genome of the newly isolated cyanobacterial strain was sequenced in this study. Based on the extracted 16S rRNA gene sequence, its phylogenetic position was analyzed (Figure 4). The cyanobacterial strain clustered well supported with the unicellular, thermophilic species *P. lividus* PCC 6715. These cyanobacteria are closely related to the genus *Thermosynechococcus* and display a more distant relationship with the genus *Synechocystis* and the species *Synechococcus elongatus* ND15, *Synechococcus elongatus* PCC 6301, *Synechococcus* sp. PCC 7918, and *Synechococcus* sp. 1002 NIES969 (Shih et al., 2013; Strunecký et al., 2023). Based on these findings and a comparison with known 16S rRNA gene sequences using the BLAST tool of GenBank (Huerta‐Cepas et al., 2019), the isolated cyanobacterial strain was categorized as a novel species within the Thermosynechococcaceae family (Figure 4). Further phylogenetic analysis based on the 16S rRNA gene assigned the strain to a separate branch, closely related to the *P. lividus* strains PCC 6715‐6717 (formerly classified as *Thermosynechococcus lividus*, *Synechococcus lividus*, or *Thermostichus lividus*) (Komárek et al., 2020).

#### 3.3.2 Whole‐genome sequence‐inferred phylogeny

**Figure A1:**
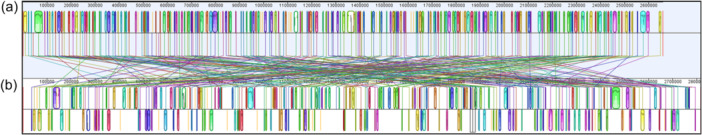
Whole‐genome alignments of (a) *Parathermosynechococcus lividus* PCC 6715 and (b) Thermosynechococcaceae cyanobacterium sp. Okahandja (this study). Alignments were performed with progressive Mauve (Koren et al., 2017), applying a Locally Collinear Block (LCB) weight of 4000 bp, and therefore only considering conserved regions with lengths above 4 kb, resulting in 212 LCBs. *T. lividus* PCC 6175 was set as reference (A, top); thus, all LCBs are depicted on its plus gene strand. Inverted genes are depicted on the minus strand (B, lower row) for the strain of this study. Corresponding LCBs are framed and colored identically, as well as connected by a line. Peaks within an LCB‐box inform about respective sequence homology, with white gaps illustrating a lack of homology.

Average nucleotide identity (ANI) analysis of the investigated strain, an established whole‐genome sequence‐based method to identify prokaryotic species (Huerta‐Cepas et al., 2017; Jarett et al., 2014), yielded maximum identities of approximately 81% with *P. lividus* PCC 6715. However, the threshold for reliable ANI‐based species identification is 95% and above, with few exceptions, for example, *Variovorax* spp. with 88%, reflecting a less distinct species relation within the genus (Seemann, 2013). At the same time, progressiveMauve (Koren et al., 2017)‐guided whole‐genome alignment with the reference strain *P. lividus* PCC 6715 (Mammoth Hot Spring, Yellowstone National Park) revealed substantial genetic differences (Figure A1), with many gene inversion events and non‐conserved regions, underpinning the role of geographical separation for gene drive. Furthermore, a digital DNA‐DNA hybridization based on a Genome Blast Distance Phylogeny (GBDP) approach was deployed on the free GGDC (genome‐to‐genome distance calculator) web service of the German Strain collection (http://ggdc.dsmz.de) (Auch et al., 2010) between the genomes of the strain of this study and *P. lividus* PCC 6715, its closest relative (Meier‐Kolthoff et al., 2013, 2022). In the process, three distinct formulas were applied to infer genome‐to‐genome distances based on obtained sets of HSPs (high‐scoring segment pairs) and MUMs (maximal unique matches). These distances were transformed to values analogous to DDH using a generalized linear model (GLM) inferred from an empirical reference data set comprising real DDH values and genome sequences. Of the three formulas, two—including the recommended formula—concluded that the genome of our study represents a distinct genome compared with that of the strain PCC 6715. Furthermore, it concluded based on the GC%‐difference of 1.53% that the two genomes belong to distinct species, since the %GC content cannot differentiate more than 1% (Meier‐Kolthoff et al., 2014). Refer to the supplementary material for details on the evaluation with GGDC: https://zenodo.org/doi/10.5281/zenodo.10007199.

#### 3.3.3 Orthology‐inferred phylogeny

**Figure 5:**
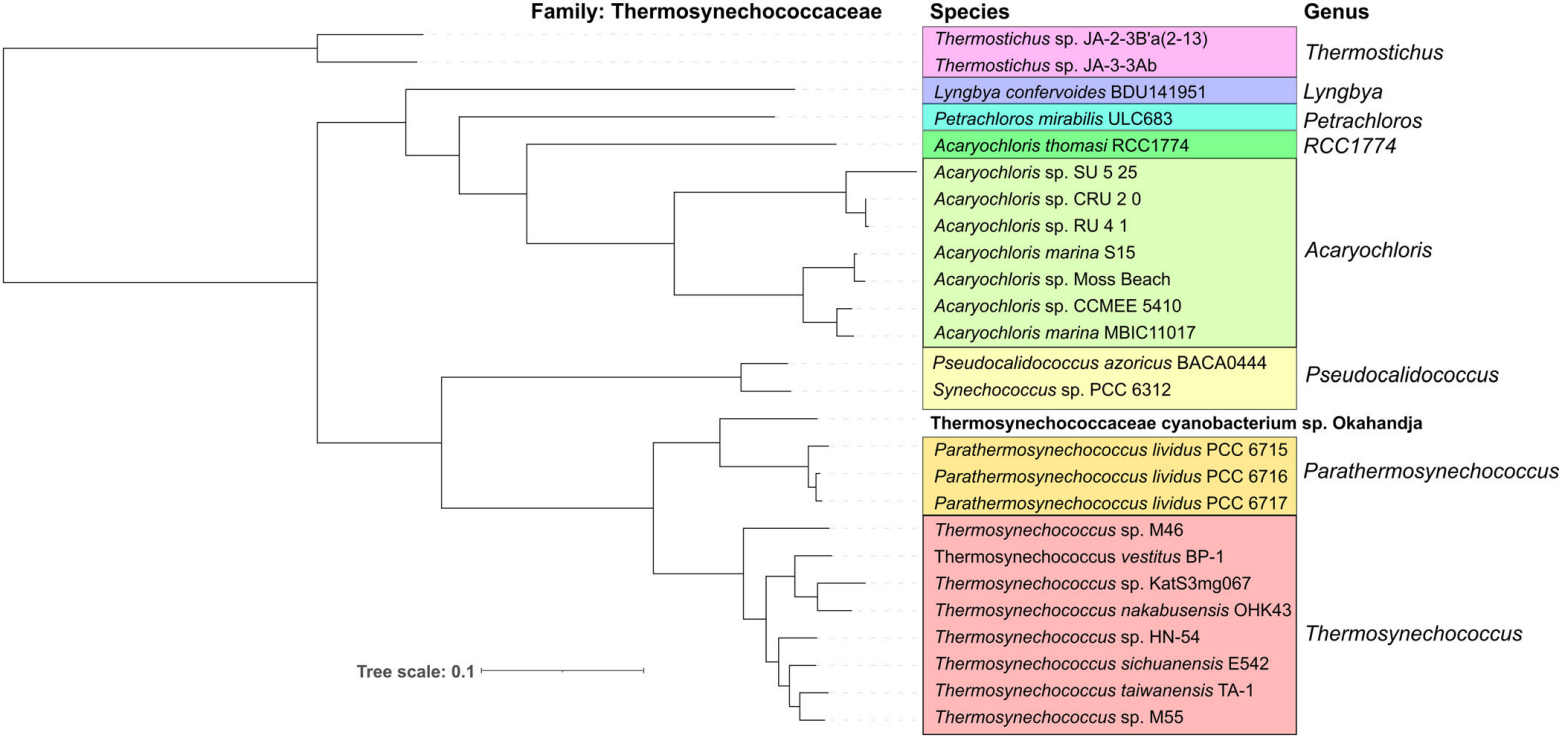
Phylogenetic tree of representative members of the family Thermosynechococcaceae based on orthologs as identified by OrthoFinder 2.5.4 (Emms & Kelly, 2015). Throughout all investigated genomes, 451 common orthogroups encompassing 270 single‐copy genes could be identified. Between the depicted Thermosynechococcus and Parathermosynechococcus genomes, 1476 shared orthogroups were detected. Gene sequence searching was performed with DIAMOND 2.0.15 (Buchfink et al., 2014), unrooted gene trees were then inferred with DendroBLAST (Kelly & Maini, 2013), and unrooted species trees were inferred with STAG (Emms & Kelly, 2018). The latter were then rooted with STRIDE (Emms & Kelly, 2017) for ortholog inference by OrthoFinder. All tools were utilized as implemented in the OrthoFinder (Emms & Kelly, 2015) pipeline on the public galaxy.eu server of the Galaxy web platform (Afgan et al., 2016). Finally, the phylogenetic species tree was visualized on iTOL (Letunic & Bork, 2024) and modified with Inkscape.

Investigation of representative Thermosynechococcaceae genomes for orthologous proteins yielded a total of 451 orthogroups (including gene duplication events) and 270 single‐copy genes (exactly one gene per species) present in all genomes (Figure 5). When only comparing the eight delineated *Thermosynechococcus* species and the three *P. lividus* strains PCC 6715, PCC 6716, and PCC 6717 according to Tang et al. (2024) with the novel strain of this study, 1476 orthogroups encompassing 1268 single‐copy genes were detected. When regarding the genomes of *Parathermosynechococcus* spp. and the novel strain from Okahandja exclusively, 2215 common orthogroups and 2090 single‐copy genes were identified. Subsequent multiple sequence alignment and phylogenetic tree construction based on the ortholog‐inferred phylogenetic tree assigned the Thermosynechococcaceae cyanobacterium sp. Okahandja of this study to a distinct and separate clade besides the Yellowstone National Park‐derived genus of *Parathermosynechococcus* (Figure 5) (Tang et al., 2024). Therefore, the orthology‐inferred phylogenetic analysis supports the results of the 16S rRNA gene sequence‐inferred phylogenetic tree (Figure 4), suggesting the existence of a novel genus comprising two species so far (strain Sagole Hotspring and Thermosynechococcaceae cyanobacterium sp. Okahandja).

#### 3.3.4 Morphological and phylogenetic characterization suggests a novel species belonging to the family of Thermosynechococcaceae

*P. lividus* PCC 6715 was formerly known as *Thermosynechococcus lividus* or *Synechococcus lividus,* belonging to the order of *Synechococcales*. Recently, it was proposed to belong to a new genus ‘*Parathermosynechococcus*’ based on GTDB classification, ANI/AAI, and phylogenomics by Tang et al. (2024). As discussed above, the newly isolated strain from this study was very closely related to the strain *P. lividus* PCC 6715 based on their 16S rRNA gene sequences. In that context, the morphological attributes of the cyanobacterial cells observed under the microscope (Figure 1) resembled previous descriptions in the literature for this species (Copeland, 1936; Tang et al., 2024). Taking into account that the investigated strain of this study was assigned to a separate branch than the strain *P. lividus* PCC 6715 based on 16S rRNA gene sequence homology and a whole‐genome sequence‐based aligned nucleotide identity (ANI) of only 81%, we propose to assign the strain of this study to the family Thermosynechococcaceae. Based on the position within the phylogenetic trees, there is an indication that this strain might belong to a novel genus. However, this hypothesis should be confirmed with more certainty if other closely related species are discovered in the future. Further data to support this view are differences in the genome‐wide GC content of 55% (this study) as opposed to 53.5% of *P. lividus* PCC 6715 and the whole‐genome sequence alignment with progressiveMauve (Koren et al., 2017) (Figures A1 and 7), which revealed dramatic genome fluctuation in the form of gene deletions, rearrangements, and inversion events. Papke et al. (2003) revealed the importance of geographic separation for allopatric speciation events in cyanobacteria using genetic marker investigation of bacterial hotspring populations worldwide (Parks et al., 2015). The authors emphasized that the hypothesis “everything is everywhere” is incorrect and that lateral gene transfer microbial community dynamics alone are not sufficient to describe speciation accurately. In accordance with many other reports (Blin et al., 2023; Darling et al., 2010; Fuchs et al., 2021; Gao et al., 2004; Hitch et al., 2021; Krzywinski et al., 2009; Manni et al., 2021; Ward et al., 2012), our data appears to confirm the thesis of Papke and colleagues.

#### 3.3.5 Phylogenetic classification according to COG

**Figure 6:**
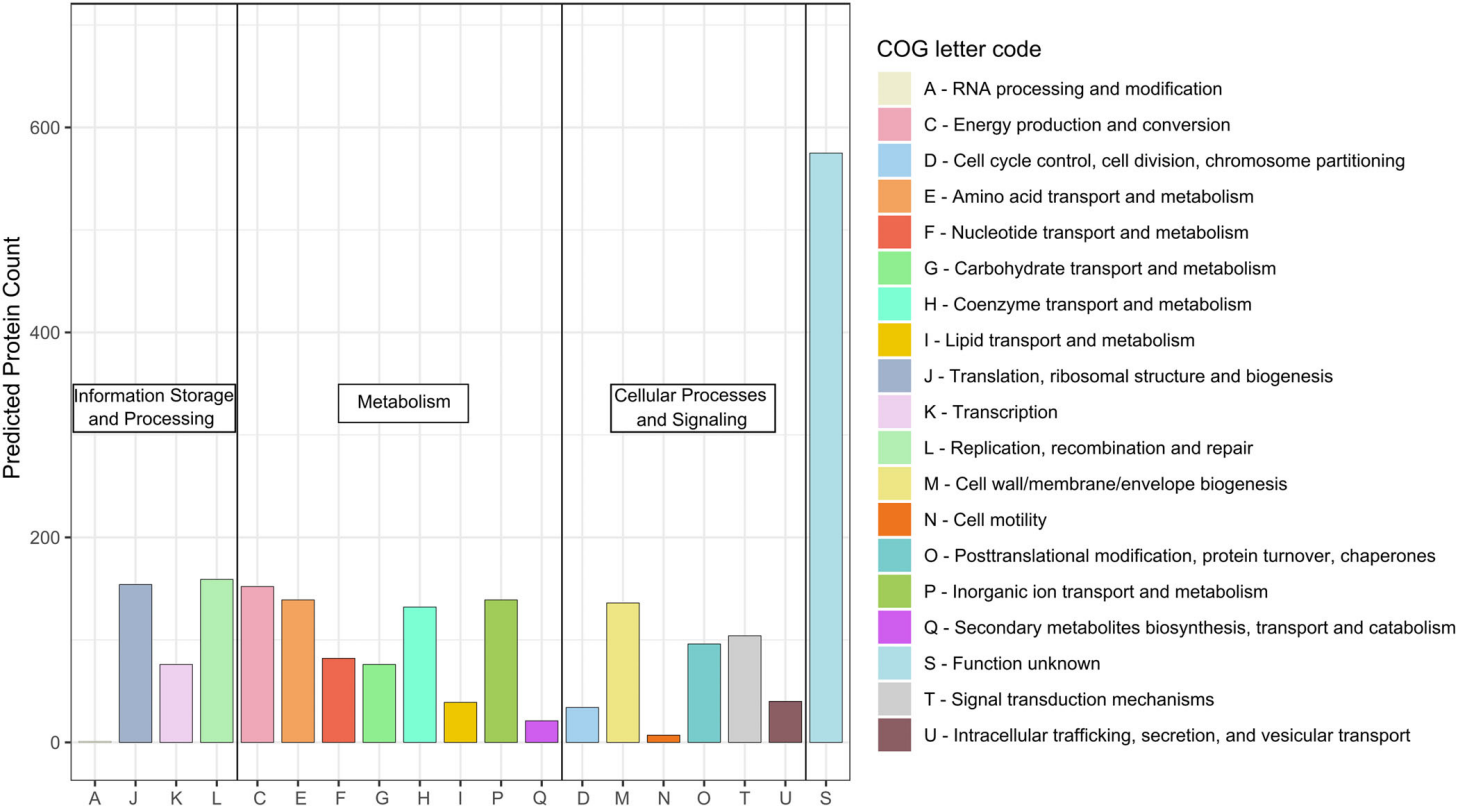
Phylogenetic classification of Thermosynechococcaceae cyanobacterium sp. Okahandja (this study) genome‐encoded proteins on the basis of the database of Clusters of Orthologous Groups of proteins (COGs). Each COG includes proteins that are inferred to be orthologs from at least three lineages. Out of the 2,162 total proteins, 575 could not be assigned a function, resulting in an annotation rate of 73.4%. COGs were obtained with eggNOG‐mapper (Kelly & Maini, 2013).

Phylogenetic classification of the genome‐encoded proteins according to the Clusters of Orthologous Groups of proteins (COGs) revealed an annotation ratio of approximately 70%, with 30% proteins of unknown function (Figure 6). Like most cyanobacteria, Thermosynechococcaceae cyanobacterium sp. Okahandja features little to no proteins involved in RNA processing and modification (A), chromatin structure and dynamics (B), extracellular structures (W), nuclear structure (Y), or a cytoskeleton (Z) (Ma et al., 2003). The general COG distribution pattern is in accordance with that of other Thermosynechococcaceae cyanobacteria, with noticeably higher fractions of translation than transcription and amino acid metabolism‐related proteins than carbohydrate metabolism‐related proteins (Adomako et al., 2022).

### 3.4 In silico analyses and visualization of the novel genome

#### 3.4.1 Whole‐genome alignment reveals profound gene flux

To investigate conserved region abundance and general genomic arrangement, a whole‐genome alignment of the closest relative, *P. lividus* PCC 6715, and Thermosynechococcaceae cyanobacterium sp. Okahandja was performed using progressiveMauve (Figure A1). Deploying a locally collinear block (LCB) weight of 4000 bp, 212 conserved regions shared among the two species could be detected. As apparent by the multitude of overlapping LCB‐connecting lines, the localization of individual conserved regions between the two aligned genomes diverges heavily. Furthermore, as indicated by the second, lower row in the depicted Thermosynechococcaceae cyanobacterium sp. Okahandja genome, about half of the conserved regions have been inverted in their respective orientation.

Finally, as evident through the numerous white gaps in between colored, LCB‐representing boxes, many genes are neither shared nor conserved between *P. lividus* PCC 6715 and Thermosynechococcaceae cyanobacterium sp. Okahandja, underpinning their genomic disparities. Considering that the genomes differ by 150 kb in size, it is physically impossible for all genetic elements to be conserved independently of their localization.

#### 3.4.2 Circular genome plot and phage region investigation

**Figure 7:**
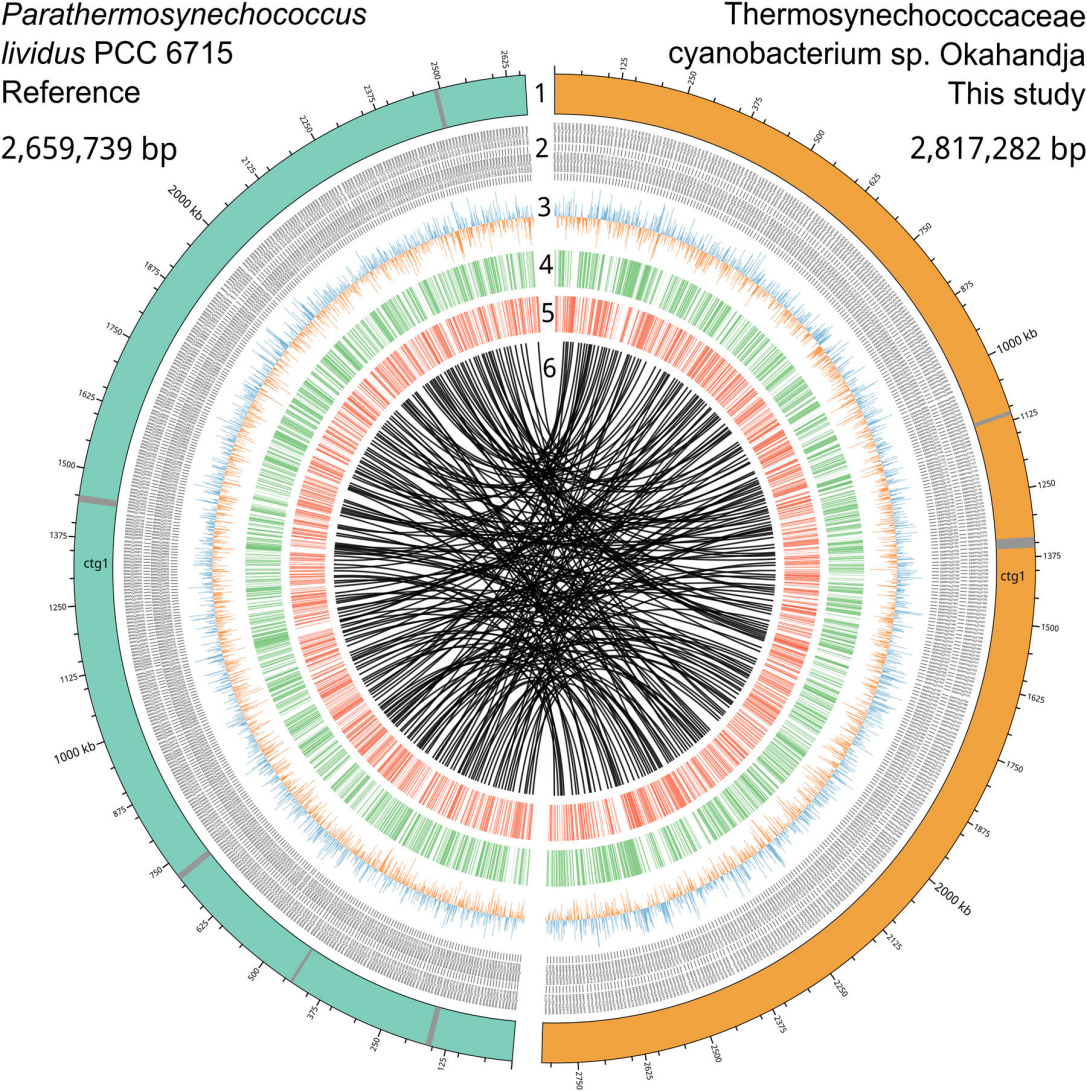
Comparative circular genome plot of Thermosynechococcaceae cyanobacterium sp. Okahandja (this study) and the reference strain *Parathermosynechococcus lividus* PCC 6715. Depicted from the outermost to the innermost circle are: (Knoll, 2008) the singular, circular contigs with ticks indicating the genome lengths and gray bands, which represent inactive phage regions, as predicted with PHASTER, (Ward et al., 2012) Genome‐encoded gene labels, (Galtier & Lobry, 1997) GC‐skews, representing increased G/C nucleotide (blue) or increased A/T nucleotide (orange) abundance compared to the genome‐wide average, (Thompson & Eisenberg, 1999) plus strand‐encoded genes, (Van Noort et al., 2013) minus strand‐encoded genes, and (Sabath et al., 2013) locally collinear blocks (LCBs) derived from whole‐genome alignment with progressiveMauve, indicating conserved regions with a LCB length of above 4000 kb.

A comparative circular plot serves to illustrate genomic similarities and disparities between Thermosynechococcaceae cyanobacterium sp. Okahandja of this study and *P. lividus* PCC 6715 (Figure 7). Since both genomes were derived from PacBio long‐read sequencing platforms, circular chromosomes could be resolved within one continuous contig via the assembly. PHASTER‐guided prediction revealed two incomplete phage regions for Thermosynechococcaceae cyanobacterium sp. Okahandja and five incomplete phage regions for *P. lividus* PCC 6715 (Team, 2022), which is indicated by the gray bands in the respective chromosomes (colored teal or orange). Visualization of the GC/AT skews uncovered balanced nucleotide distributions throughout the whole cyanobacterial genomes, in contrast to those of many bacteria, where regions of GC or AT overabundance persist (Wickham, 2008). The innermost circle depicts the results from the progressiveMauve whole‐genome alignment (Koren et al., 2017) (Figure A1), emphasizing the multitude of conserved regions (origin of lines), their genomic rearrangement (lines), and non‐conserved regions (white gaps).

#### 3.4.3 Thermosynechococcaceae cyanobacterium sp. Okahandja harbors three biosynthetic gene clusters for terpenes

**Figure 8:**
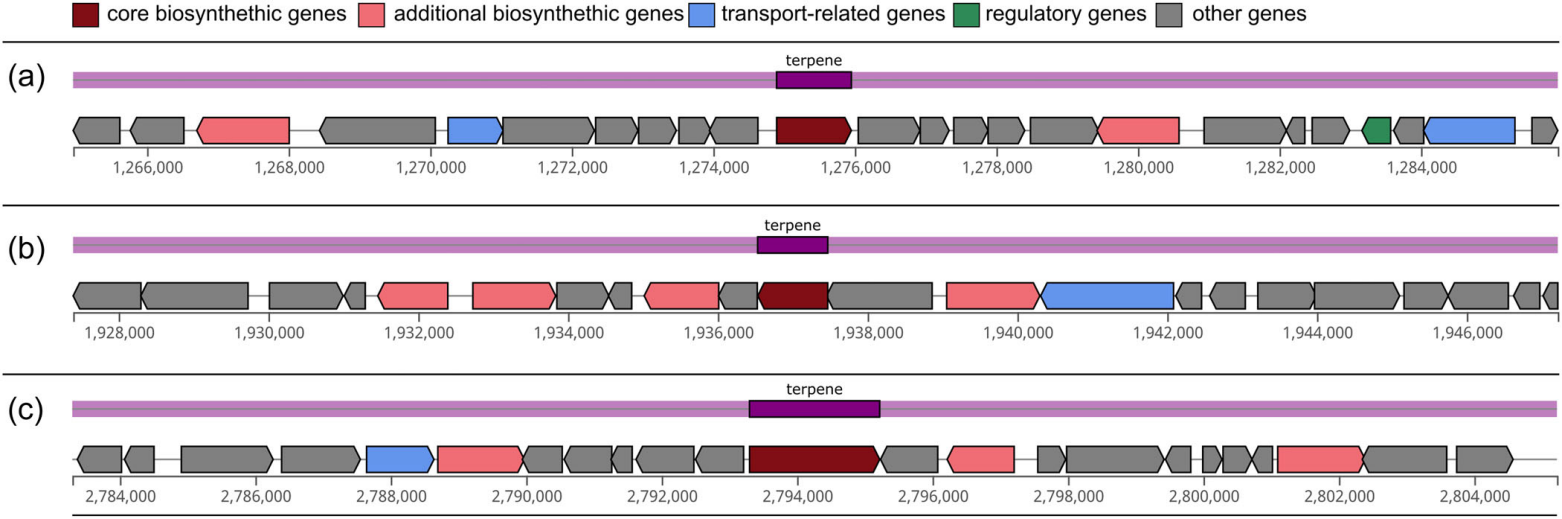
Biosynthetic gene clusters (BCGs) of Thermosynechococcaceae cyanobacterium sp. Okahandja (this study) predicted with antiSMASH (Blin et al., 2023). Three BCGs, all of which are involved in terpene biosynthesis, could be identified and localized within the genome: (a) Cluster I spans over 20,977 nucleotides on the plus strand (bases 1,274,895–1,275,951) and includes the genes 1208–1232. The biosynthetic core gene 1219 is annotated as 6‐carboxytetrahydropterin synthase. (b) Cluster II spans over 19,823 nucleotides on the minus strand (bases 1,936,532–1,937,468) and includes the genes 1843–1864. The biosynthetic core gene 1853 is annotated as phytoene synthase. (c) Cluster III spans over 21,923 nucleotides on the plus strand (bases 2,793,297–2,795,220) and includes the genes 2687–2710. The biosynthetic core gene 2698 is annotated as squalene‐hopene cyclase.

Inquiry for industrially relevant secondary metabolite production genes with antiSMASH 7.0 (Blin et al., 2023) revealed three terpene‐producing biosynthetic gene clusters (BCGs) of approximately 20 kbp length, harboring one core gene each (Figure 8). Hereby, the terpene synthesizing proteins of the BCGs I–III were annotated as 6‐carboxytetrahydropterin synthase, phytoene synthase, and squalene‐hopene cyclase, respectively. Opposed to many cyanobacteria that belong to more recent evolutionary clades, no BCGs for non‐ribosomal peptide, bacteriocin, or polyketide production could be detected in our investigated strain (Ma et al., 2003). Phytoene is a colorless carotenoid intermediate, exhibiting an unusually low number of three conjugated double bonds and acting as a precursor for other carotenoids (Strunecký et al., 2023). Consequently, phytoene absorbs light at the UVB region (280–320 nm) and is structurally less rigid. Implications are potentially photoprotective features against UV‐B light‐induced skin cancers (Shih et al., 2013) and a significantly higher bioaccessibility (Strunecký et al., 2023), partly because aggregation and crystal formation are less likely (Camacho et al., 2009). Data also suggest that the colorless carotenoids play a more important role in nutrition than previously thought, with intake amounts exceeding those of the major dietary carotenoids lycopene, lutein, β‐cryptoxanthin, violaxanthin, neoxanthin, and zeaxanthin (Komárek et al., 2020). Therefore, the phytoene synthase of Thermosynechococcaceae cyanobacterium sp. Okahandja might represent an interesting candidate for recombinant carotenoid production.

#### 3.4.4 Insights from inspection of the CRISPR/Cas machinery

Functionally, the CRISPR (clustered regularly interspaced short palindromic repeats)‐Cas (CRISPR‐associated) adaptive immune system acts as a bacterial defense mechanism to deflect invading mobile elements, including phage attacks (Westra et al., 2016). Jahodářová et al. examinated the CRISPR/Cas machinery evolution in *Thermostichus* spp. (Jahodářová et al., 2022), which uncovered differences in CRISPR spacer diversity based on geographically exclusive phage predation pressure. Spacer regions are phage genome‐derived sequences incorporated by singular bacteria cells into their CRISPR arrays to acquire resistance against that specific bacteriophage (Barrangou et al., 2007), a phenotype that is inherited by future clones (Koonin & Wolf, 2009; Wang et al., 2016; Westra et al., 2016). Following that example, we inquired about the novel genome for CRISPR‐associated genes, which are more abundant and longer among thermophiles (Anderson et al., 2011), revealing 19 genome‐encoded proteins with eggNOG‐mapper (Huerta‐Cepas et al., 2019) and 18 with RAST (Aziz et al., 2008; Brettin et al., 2015; Overbeek et al., 2014), belonging to class 1 (Jahodářová et al., 2022; Westra et al., 2016). Contrarily, the closest relative, *P. lividus* PCC 6715, which originated from the Octopus Spring in the Yellowstone National Park (Dyer & Gafford, 1961), exhibited merely two CRISPR‐associated genes over 2 arrays, encompassing 10 spacers and 12 repeats (Table 1). The second member of the genus *Parathermosynechococcus (*Tang et al., 2024 *)*, strain PCC 6717, which was also sampled in the Yellowstone National Park (Fairy Spring), exhibited similarly low CRISPR‐associated gene numbers with 9 over 3 arrays, encompassing 22 spacers and 25 repeats. The third specimen, *P. lividus* PCC 6716, was derived from the Hunter's Hot Spring in Oregon and has a comparably extensive CRISPR/Cas system as the investigated strain from Namibia. Genome annotation with RAST (Aziz et al., 2008; Brettin et al., 2015; Overbeek et al., 2014) revealed a total of 19 CRISPR‐associated genes, 69 spacers, and 74 repeats distributed over 5 arrays.

**Table 1:** CRISPR and CRISPR‐associated (Cas) gene elements in the genomes of the *Parathermonsynechococcus lividus* strain PCC 6715‐17 and the strain of this study, Thermosynechococcaceae cyanobacterium sp. Okahandja as annotated by RAST.

Strain	Arrays	Genes	Repeats	Spacers	Origin	Year
PCC 6715	2	2	12	10	Octopus Spring, Yellowstone National Park	1961
PCC 6716 OH‐53s	5	19	74	69	Hunter's Spring, Oregon	1962
PCC 6717 Y52s	3	9	25	22	Fairy's Spring, Yellowstone National Park	1964
Okahandja	6	18	137	131	Hotspring near Okahandja, Namibia	2024

CRISPR spacer sequence alignments of Thermosynechococcaceae cyanobacterium sp. Okahandja with *P. lividus* PCC 6715 revealed no shared spacer regions, confirming yet again that CRISPR arrays target local rather than global phages (Kunin et al., 2008) and that the antagonistic host–virus coevolution outpaces bacterial dispersal (Berg Miller et al., 2012; Westra et al., 2016). Furthermore, a nucleotide BLAST search of all CRISPR spacer sequences resulted in no matches with virus genome sequences, which is a common phenomenon (Anderson et al., 2011). This could be attributed to CRISPR/Cas targeting rare viruses only (Emerson et al., 2013) but more likely due to the limited accessibility of phage sequences (Andersson & Banfield, 2008; Westra et al., 2016).

Analogous to the thermophilic PCC 6715, the two *Thermostichus* strains JA2‐3B'a (Abed et al., 2009; Burra et al., 2010; Fish & Codd, 1994; Galtier & Lobry, 1997; Graverholt & Eriksen, 2007; Kumar et al., 2019; Nishioka et al., 2001; Patel et al., 2019; Sabath et al., 2013; Thompson & Eisenberg, 1999; Van Noort et al., 2013; Ward et al., 2012) and 3‐3Ab were derived from the Octopus Spring (Yellowstone National Park) (Allewalt et al., 2006). Interestingly, the analysis of Jahodářová et al. revealed far more extensive CRISPR/Cas machinery for the two *Thermostichus* spp. compared to PCC 6715 despite their identical habitat (Jahodářová et al., 2022). The extreme time discrepancy between the sample collection of PCC 6715 in 1961 (Dyer & Gafford, 1961) and the two *Thermostichus* specimens in 2006 may serve to explain the divergent phage predation pressures and provide evidence for the continuous coevolution.

The importance of the CRISPR immune response in phage attacks is further illustrated in the increased prophage sequence count (dormant, non‐lytic phage sequences) in the genome of PCC 6715 (five, refer to Figure 7) as opposed to merely one prophage sequence in Thermosynechococcaceae cyanobacterium sp. Okahandja, the latter with a more complex CRISPR/Cas system at its disposal. A possible conclusion would be that the various horizontal gene transfer (HGT) events, visualized by the whole‐genome sequence alignment with progressiveMauve (Darling et al., 2010), happened to PCC 6715 but not to the better‐defended strain evaluated in this study, implying that Thermosynechococcaceae cyanobacterium sp. Okahandja is genetically closer to the common progenitor. It has been demonstrated that the CRISPR/Cas system can also act as an HGT barrier (Erdmann & Garrett, 2012; Palmer & Gilmore, 2010), although contradicting evidence has been reported (Gophna et al., 2015; Westra et al., 2016), as well. However, the focal point of this hypothesis is the time point of CRISPR/Cas system acquirement or loss thereof, depending on which of the two scenarios holds true.

### 3.5 Differential proteome analysis uncovers 129 heat‐dependent proteins

**Figure 9:**
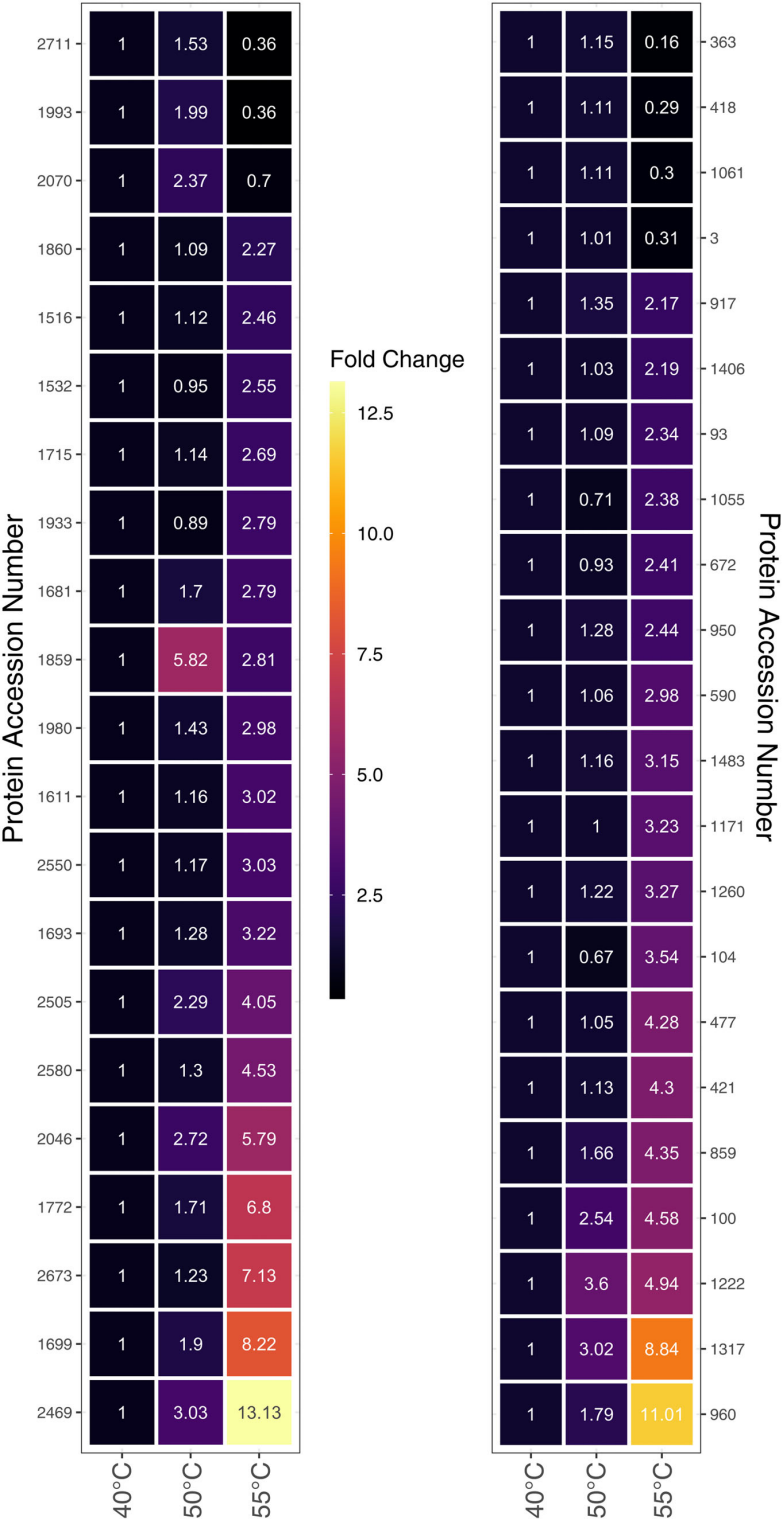
Heatmaps of differentially expressed proteins in Thermosynechococcaceae cyanobacterium sp. Okahandja (this study) at 40°C, 50°C, or 55°C. For each temperature condition, nine technical samples were taken and evaluated. Detected expression levels were normalized with the 40°C sample; thus, all depicted fold‐change counts at 50 or 55°C refer to the respective expression level at 40°C.

Evaluation of the intracellular Thermosynechococcaceae cyanobacterium sp. Okahandja proteomes derived from cultivations conducted at 40°C, 50°C, or 55°C revealed 43 upregulated proteins at elevated temperatures (Figure 9). Overall, if one includes the proteins with reduced expression levels at elevated temperatures, 129 differentially expressed, thermosensitive proteins could be identified (supplementary material for the complete list: https://zenodo.org/doi/10.5281/zenodo.10007199). It is important to note that samples were normalized based on the 40°C controls; thus, all fold changes at 50°C or 55°C are depicted with respect to protein abundancies at the lowest temperature condition. To that end, seven proteins showed increased abundances at 50°C, exclusively, followed by decreased expression levels at 55°C (Gene IDs 3, 363, 418, 1061, 1993, 2070, 2711). Additionally, 17 proteins were 1.2‐fold (or 20%) more abundant at 55°C, but not at 50°C, compared to the 40°C condition (gene IDs 93, 104, 153, 421, 477, 590, 672, 1055, 1171, 1406, 1483, 1516, 1611, 1715, 1860, 1933, and 2550). A singular protein (gene ID 1859) was upregulated at both elevated temperatures, with a maximum abundance at 50°C. The remaining 18 proteins (gene IDs 100, 859, 917, 950, 960, 1222, 1260, 1317, 1681, 1693, 1699, 1772, 1980, 2046, 2469, 2505, 2580, 2673) were increasingly upregulated with elevated temperature, reaching fold changes of up to 13 at 55°C.

Detailed inspection of the 10 topmost upregulated enzymes at elevated temperatures (Table 2) uncovered three hypothetical proteins, two of which could be annotated as nuclease activity containing protein (gene ID 2469) and signal transduction histidine kinase (gene ID 960), respectively, with eggNOG‐mapper 5.0 (Huerta‐Cepas et al., 2019). Other proteins included a dTDP‐4‐dehydrorhamnose 3,5‐epimerase (gene ID 1699), an ergothioneine biosynthesis protein EgtC (gene ID 1859), a Ycf51 family protein (gene ID 1222), a type II toxin–antitoxin system VapC family toxin (gene ID 1772), a crossover junction endodeoxyribonuclease RuvC (gene ID 2046), the 50S ribosomal protein L33 (gene ID 2673), and a carbohydrate ABC transporter permease (gene ID 100). Except for the ergothioneine biosynthesis protein EgtC, all proteins were more abundant at 55°C than at 50°C with fold changes between approximately 1.4‐fold for the Ycf51 family protein and sixfold for the putative signal transduction histidine kinase.

**Table 2:** The 10 topmost differentially upregulated proteins of Thermosynechococcaceae cyanobacterium sp. Okahandja in dependence of temperature. Log2 fold changes (Log2 FC) refer to expression levels at 40°C. Annotations were performed by NCBI's Prokaryotic Genome Annotation Pipeline (Haft et al., 2018; Li et al., 2021; Tatusova et al., 2016), which utilizes the TIGRFAM (Haft et al., 2013) database and eggNOG‐mapper (Huerta‐Cepas et al. 2017, 2019), deploying the eggNOG 5.0 database.

Gene ID	Log2 FC (50°C)	Log2 FC (55°C)	Annotation		
			PGAP	eggNOG	KEGG
2469	3,03	13,13	Hypothetical protein	Nuclease activity	No hits
960	1,79	11,01	Hypothetical protein	Signal transduction histidine kinase	No hits
1317	3,02	8,84	Hypothetical protein	No hits	No hits
1699	1,9	8,22	dTDP‐4‐dehydro‐rhamnose 3,5‐epimerase	Catalyzes the epimerization of the C3′ and C5′ positions of dTDP−6‐deoxy‐d‐xylo‐4‐hexulose, forming dTDP‐6‐deoxy‐l‐lyxo‐4‐ hexulose	rfbC, rmlC; dTDP‐4‐dehydro‐rhamnose 3,5‐epimerase
1859	5,82	2,81	Ergothioneine bio‐synthesis protein EgtC	TIGR03442 family protein (ergothioneine biosynthesis protein EgtC)	No hits
1222	3,6	4,94	Ycf51 family protein	PFAM Protein of function (DUF2518)	No hits
1772	1,71	6,8	Type II toxin–antitoxin system VapC family toxin	Large family of predicted nucleotide‐binding domains	No hits
2046	2,72	5,79	Crossover junction endodeoxyribonu‐clease RuvC	Nuclease that resolves Holliday junction intermediates in genetic recombination. Cleaves the cruciform structure in supercoiled DNA by nicking to strands with the same polarity at sites symmetrically opposed at the junction in the homologous arms and leaves a 5′‐terminal phosphate and a 3′‐terminal hydroxyl group	ruvC; crossover junction endo‐deoxy‐ribonuclease RuvC
2673	1,23	7,13	50S ribosomal protein L33	Belongs to the bacterial ribosomal protein bL33 family	RP‐L33, MRPL33, rpmG; large subunit ribosomal protein L33
100	2,54	4,58	Carbohydrate ABC transporter permease	ABC‐type sugar transport system, permease component	chiG; putative chitobiose transport system permease protein

#### 3.5.1 Adaptation of protein expression patterns to elevated temperatures

Please refer to Table A2 in the appendix for a curated list of all discussed proteins in the following sections.

##### Modulation of the genetic reproduction machinery

RNAs and proteins involved in transcription and translation possess higher GC contents in thermophiles under elevated temperatures (Basak et al., 2004), but high genome GC compositions alone are no indicators of thermophily or at least not universally applicable (Basak et al., 2004; Wang et al., 2015; Zeldovich et al., 2007). With 57.3%, the average GC content of all 41 genomic encoded transfer RNAs, detected with tRNAscan (Lowe & Eddy, 1997), was indeed slightly above the genome‐wide average GC distribution of 55%.

Among the 129 differentially expressed thermosensitive proteins, 13 were associated with gene transcription or translation. Of these, three proteins with nuclease activity (gene IDs 2046, 2469 & 2574) were identified, where two proteins were incrementally upregulated (gene IDs 2046 & 2469), while the remaining nuclease was incrementally downregulated with increasing temperature. While protein 2469 is annotated as nuclease by eggNOG‐mapper based on sequence homology, 2046 is the crossover junction endodeoxyribonuclease RuvC involved in homologous recombination. Interestingly, RuvC endonuclease‐like domain containing CRISPR/Cas class 2 effectors have been identified, suggesting a potential involvement in the adaptive immune system of protein 2469 (Mapelli‐Brahm & Meléndez‐Martínez, 2021). The last nuclease of the identified triplet was the exoribonuclease D downregulated 2.7‐2.9‐fold with increasing temperature and functionally relevant for tRNA maturation (Zuo et al., 2005).

Additional incrementally upregulated proteins associated with genetic reproduction encompass the polymerase III subunit delta (gene ID 1611) responsible for the high speed and processivity of the polymerase III (Jeruzalmi et al., 2001), which is in turn involved in DNA replication, homologous recombination, and mismatch repair according to the KEGG classification (Kanehisa et al., 2016); the 50S ribosomal protein L33 (gene ID 2673), and the phenylalanyl‐tRNA synthetase beta chain pheT (gene ID 421).

In contrast, the following five proteins were downregulated incrementally with increasing temperature: the segregation and condensation protein A, relevant for chromosomal partition during cell division (Mascarenhas et al., 2002; Soppa et al., 2002); the 30S ribosomal protein S12 (gene ID 158), involved in 30S and 16S rRNA stabilization (Demirci et al., 2013); the polyribonucleotide nucleotidyltransferase (gene ID 594), involved in mRNA degradation (Kleppe et al., 1971); the DNA‐3‐methyladenine glycosylase (gene ID 2513), responsible for base excision repair (Wyatt et al., 1999); and the DNA adenine methylase (gene ID 2324), realizing mismatch repair (Low et al., 2001). According to eggNOG‐mapper (Huerta‐Cepas et al., 2017; Huerta‐Cepas et al., 2019), second putative chromosome segregation enforcing protein (gene ID 2711) was detected to be upregulated 1.5‐fold at 50°C and then downregulated 2.8‐fold at 55°C. In addition, a third ribosome‐associated protein, the translation inhibitor RaiA (gene ID 2297), was steadily downregulated approximately threefold at both 50°C and 55°C. It is required for dimerization prevention of 70S ribosomes in the stationary phase (Ueta et al., 2005), reduces translation errors (Ueta et al., 2005), and inactivates 70S ribosomes during environmental stress in a reversible manner (Lang et al., 2021). Finally, a pentapeptide repeat‐containing protein (gene ID 418) of unknown function, predicted to bind single‐stranded DNA (Huerta‐Cepas et al., 2017; Huerta‐Cepas et al., 2019), was downregulated −3.5‐fold at 55°C, exclusively.

While ribosomal proteins have been reported to be more abundant at high temperatures before, for example, in *Bacillus methanolicus* MGA3 and *Pyrococcus furiosus* (Müller et al., 2014; Trauger et al., 2008), the upregulation of the 50S ribosomal protein L33 is curious, as it does not have any significant role in ribosome synthesis or assembly in *E. coli* (Maguire et al., 1997). It could be hypothesized that either (i) L33 does play a pivotal role in the ribosome synthesis and assembly of Thermosynechococcaceae cyanobacterium sp. Okahandja acts as a physical “heat shield” for other critical cell parts or (ii) it is a side effect of the whole ribosomal protein operon being transcribed. Moreover, ribosomal protein complexes in thermophiles are reported to have stronger binding to 23S rRNA and more compact structures compared to those in mesophiles (Shcherbakov et al., 2006), which might secure the biosynthesis of functional proteins under adverse temperature conditions (Wang et al., 2015).

#### 3.5.2 Differential expression of regulatory and CRISPR/Cas proteins

Closer inspection of regulatory proteins revealed a total of 11 proteins with expression levels depending on temperature conditions. Nine of these regulators were downregulated, whereas only two regulatory proteins were upregulated, namely a two‐component system belonging to protein pixH required for phototactic motility in *Synechocystis* (Yoshihara & Ikeuchi, 2004), which was upregulated threefold (gene ID 1693) at 55°C, and a signal transduction histidine kinase (gene ID 960), which was upregulated 16‐fold at the highest temperature. This histidine kinase might act as a global heat response regulator molecule, similar to σ^32^ in *E. coli* (Browning & Busby, 2004), paired with the 4.5‐fold downregulated heat‐inducible transcriptional repressor HrcA (gene ID 2557) controlling the transcription of class I heat shock genes (grpE‐ dnaK‐dnaJ and groELS operons). Protein expression and characterization or knock‐out experiments would have to confirm or deny the role of the histidine kinase in the thermal response.

AYcII family protein (gene ID 652) found to be fused to the RNA polymerase sigma‐70 factor family domain and therefore possibly involved in transcription initiation (Yeats et al., 2003) and a YbaB/EbfC family nucleoid‐associated protein (gene ID 1449) were downregulated the most, with 25‐ and 16‐fold at 55°C, respectively. According to eggNOG‐mapper (Huerta‐Cepas et al., 2017; Huerta‐Cepas et al., 2019), the latter binds to DNA and alters its conformation and may additionally be involved in the regulation of gene expression, nucleoid organization, and DNA protection. Interestingly, the third most downregulated protein at 55°C with eightfold was an ATP‐binding histidine kinase (gene ID 302), annotated as phosphate regulon sensor histidine kinase PhoR by KEGG (Kanehisa et al., 2016). Other incrementally less abundant proteins with increasing temperature encompassed a GGDEF domain‐containing protein (gene ID 1885), acting as diguanylate cyclase and cyclic di‐GMP synthase (Paul et al., 2004; Ryjenkov et al., 2005); an ArsR/SmtB family metalloregulator (gene ID 1377), a LysR family transcriptional regulator (gene ID 1647); and the circadian clock protein KaiA (gene ID 1886). Finally, a translation elongation factor 4 protein (gene ID 2313), annotated as GTP‐binding protein LepA, required for accurate and efficient protein synthesis under certain stress conditions, was downregulated approximately 3.8‐fold at 50°C and 1.7‐fold at 55°C.

Three temperature‐dependent CRISPR/Cas proteins could be detected in the proteomic data set. However, no clear trend for a definitive up‐ or downregulation of the adaptive immune response proteins could be identified. To elucidate, an endonuclease Cas1 (gene ID 1513) was incrementally downregulated with increasing temperature, while a type I‐E CRISPR‐associated protein Cas7/Cse4/CasC (gene ID 1993) was upregulated at 50°C and downregulated at 55°C and finally a type III‐B CRISPR‐associated protein Cas10/Cmr2 (gene ID 1516), which was incrementally upregulated with rising temperature.

##### Potential role of a toxin–antitoxin system for thermal resistance

Bacterial type II toxin–antitoxin systems, a component of which was upregulated almost sevenfold at 55°C, have been reported before to convey heat resistance, for example, the ParDE system in Enterococcaceae (Kamruzzaman & Iredell, 2019) or in the hyperthermophilic crenarchaeon *Sulfolobus solfataricus* (Polack et al., 2020). Toxin–antitoxin operons are a prevalent genetic feature found in bacteria (Pandey & Gerdes, 2005). These operons consist of two distinct genes: one encoding a harmful toxin and the other encoding a fragile antidote molecule (Kamruzzaman et al., 2021). In general, when transcription and translation processes are active, these genes produce proteins that interact to form a harmless complex. However, if the transcription or translation of the toxin–antitoxin operon is interrupted, the unstable antitoxin protein undergoes degradation through various mechanisms. This degradation leaves the toxin unchecked, allowing it to exert its biological impact (Overbeek et al., 2014). This impact can manifest as either killing the bacteria (bactericidal), inhibiting bacterial growth (bacteriostatic), or inducing a dormant metabolic state (Magnuson, 2007; Tsilibaris et al., 2007). The biological role and significance of bacterial toxin–antitoxin operons have sparked a wide array of hypotheses. These range from considering them as simple self‐interested genetic elements disseminated through horizontal gene transfer to functioning as regulators of metabolic processes within the bacterial cell (Kamruzzaman et al., 2021; Magnuson, 2007; Tsilibaris et al., 2007).

In that regard, protein modeling with SwissModel (Studer et al., 2020; Waterhouse et al., 2018) yielded the highest score for a hypothetical protein PAE0151 of the hyperthermophilic archeon *Pyrobaculum aerophilum* localized in a PIN‐domain toxin–antitoxin operon (*vapBC* operon) (Bunker et al., 2008). Additionally, the authors could demonstrate that PAE0151 (putative antitoxins) and adjacent PIN‐domain proteins (putative toxins) are conserved in many thermophilic species, indicating their involvement in the heat response (Bunker et al., 2008).

##### Expression of photosynthesis‐related proteins is attenuated during elevated temperatures

Investigation of the heat‐dependent differentially expressed photosynthesis‐related proteins suggests a strong trend toward downregulation at elevated temperatures. The data specifically indicated that of the nine detected proteins, all but one exhibited incremental downregulation with increasing temperature. In this context, it is noteworthy that the phycobilisome rod‐core linker polypeptide (gene ID 1483) stood as a unique exception (Glauser et al., 1992). This photosynthesis‐related protein displayed incremental upregulation, with fold changes of 1.15 and 3.15 at 50°C and 55°C, respectively. All other proteins, including the photosystem I reaction center subunit II (gene ID 694), the chlorophyll a‐b binding domain‐containing protein (gene ID 1748), the photosystem II manganese‐stabilizing polypeptide (gene ID 1572), the photosynthetic/respiratory NAD(P)H‐quinone oxidoreductase subunit C (gene ID 1413), the apocytochrome f (gene ID 2629), the phycocyanobilin lyase subunit beta (gene ID 1482), the protoporphyrinogen oxidase (gene ID 623), and the chlororespiratory reduction protein 7 (gene ID 697) were downregulated between 2.2‐ and ninefold at 55°C, respectively. Hence, attenuation of photosynthesis was corroborated by the proteomic data set.

##### Differential expression of transport, ion homeostasis, and redox‐related proteins

Examining transport‐related proteins in dependence of temperature revealed incremental downregulation of a cobalt/nickel importer (gene ID 2077), a lipopolysaccharide exporter (gene ID 506), a cysU, sulfate/thiosulfate transport system permease protein (gene ID 1624), the protein translocase subunit secF (gene ID 710), and a multicomponent Na + /H+ antiporter subunit B (gene ID 908). Contrarily, a SLC13 permease (gene ID 1681), annotated as Na + /H+ antiporter NhaD/arsenite permease‐like protein by eggNOG‐mapper (Huerta‐Cepas et al., 2017; Huerta‐Cepas et al., 2019), and a chitobiose import protein chiG (gene ID 100) were found to be incrementally upregulated with rising temperature, 3.8‐ and 4.6‐fold at 55°C, respectively. Finally, the signal protein processing leader peptidase SppA (gene ID 1171) was upregulated threefold at 55°C, exclusively.

A closer inspection of ion homeostasis revealed a complex heat response expression pattern, with six protein abundancies correlating with temperature changes. Three proteins, among them a ferrous iron transport protein A (gene ID 1055), were upregulated at 55°C, exclusively, with the ferrous ion transport protein slightly downregulated at 50°C. The other two proteins were a SUMF1/EgtB/PvdO family nonheme iron enzyme (gene ID 1860), annotated as **f**ormylglycine‐generating sulfatase enzyme by eggNOG‐mapper (Huerta‐Cepas et al., 2017; Huerta‐Cepas et al., 2019), and a 2Fe–2S iron‐sulfur cluster‐binding protein (gene ID 93).

In contrast, two proteins, including a 2Fe–2S iron‐sulfur cluster‐binding protein (gene ID 2641) and a hypothetical protein (gene ID 1190), both annotated as ferredoxin by eggNOG‐mapper (Huerta‐Cepas et al., 2017; Huerta‐Cepas et al., 2019), were downregulated incrementally with increasing temperature, 6.7‐fold and threefold, respectively, at 55°C. Finally, a ferredoxin thioredoxin reductase variable alpha chain (gene ID 2070) was found to be upregulated approximately 2.7‐fold at 50°C but downregulated 1.4‐fold at 55°C.

Additionally, several redox protein levels were affected by heat, comprising the NADH‐quinone oxidoreductase subunit J (gene ID 363) required for the respiratory chain (Verkhovsky & Bogachev, 2010), which was marginally upregulated at 50°C but downregulated sixfold at 55°C. Furthermore, a BCP, PRXQ, DOT5 thioredoxin‐dependent peroxiredoxin, which catalyzes the reduction of H_2_O_2_ and organic hydroperoxides (Nelson et al., 2011; Reeves et al., 2011), and an AarF/ABC1/UbiB kinase family protein (gene ID 553), associated with ubiquinone biosynthesis in *E. coli* (Poon et al., 2000) or 2′‐*N*‐acetyltransferase in *Providencia stuartii* (Macinga et al., 1998), could be detected. Both enzymes were incrementally downregulated with increasing temperature, with fold changes of −7.7 and −4.4, respectively, at 55°C. Last but not least, a peptide methionine sulfoxide reductase MsrA (gene ID 2402), involved in the reversible oxidation–reduction of methionine sulfoxide in proteins to methionine and oxidative damage repair of proteins (Weissbach et al., 2002), was increasingly downregulated 1.35‐ and 4.6‐fold at 50°C and 55°C, respectively. Consequently, Thermosynechococcaceae cyanobacterium sp. Okahandja seems to be more susceptible to oxidative stress with diminished oxidative respiration capabilities in exchange for increased fitness at elevated temperatures.

##### Metabolic adaptations to heat stress

When inspecting general metabolic proteins, we could identify 15 proteins with varying abundance depending on temperature (Table A2). Epitomizing the heavy metabolic burden on Thermosynechococcaceae cyanobacterium sp. Okahandja, 13 of these proteins were incrementally less biosynthesized at 50°C and subsequently 55°C normalized to expression levels at 40°C. While a GNAT family *N*‐acetyltransferase (gene ID 104) transferring the acetyl residue from acetyl‐coenzyme A onto diverse substrates (Shirmast et al., 2021) was upregulated 3.5‐fold at 55°C and downregulated 1.5‐fold at 50°C, solely a fructosamine kinase (Gemayel et al., 2007) (gene ID 917) was upregulated incrementally with rising temperature, 1.35 and 2.2‐fold, respectively. The opposite was true for all other identified metabolic proteins, including the isoleucine‐generating citramalate synthase (gene ID 955) (Howell et al., 1999); the glucose‐6‐isomerase (gene ID 2040), catalyzing the reversible isomerization of glucose‐6‐phosphate to fructose‐6‐phosphate (Hansen et al., 2001); and the chorismate mutase (gene ID 1170), which generates prephenate from chorismate in the shikimate pathway of tyrosine and phenylalanine biosynthesis (Hur & Bruice et al., 2003).

Interestingly, the most downregulated protein with 25‐fold at 55°C is a squalene‐hopene/tetraprenyl‐beta‐curcumene cyclase shc (gene ID 2698) according to KEGG (Kanehisa et al., 2016), which was also identified during our biosynthetic gene cluster analysis. The protein shows sequence similarity with a squalene‐hopene cyclase (Hoshino et al., 2004; Sato et al., 2004) and a bifunctional triterpene/sesquiterpene cyclase (Sato et al., 2011a, 2011b), both products of which are not linked to heat resistance.

The second and third most downregulated proteins were a TldD/PmbA family protein (gene ID 1725), functioning as peptidase or *N*‐deacetylase (Vobruba, 2022), which was downregulated first 6‐fold and then 14‐fold with increasing temperature, and the essential enzyme phosphoribosylglycinamide formyltransferase (gene ID 2289). It is required for the third step in purine biosynthesis (Aimi et al., 1990; Mullen & Jennings, 1996) and was downregulated approximately twofold at 50°C and 11‐fold at 55°C. Although Thermosynechococcaceae cyanobacterium sp. Okahandja grew comparably well at 55°C compared to 50°C in our experiments, a long‐term shortage of purines would drastically hamper the cell's genetic replication machinery. Even more so given their high abundance in mRNA of thermophilic organisms (Paz et al., 2004).

Further incrementally downregulated proteins with temperature increase included an M23 family metallopeptidase (gene ID 190), targeting peptidoglycans of the cell wall (Razew et al., 2022); two glycosyltransferase family 1 proteins (gene ID 2427 & 2708), transferring glycosyl moieties from activated sugar molecules to diverse acceptors (Zhang et al., 2020); a haloacid dehalogenase superfamily, subfamily IA (gene ID 1135); a deoxyhypusine synthase (gene ID 2533), required for homospermidine biosynthesis and biazotrophic growth in *Anabaena* spp. (Burnat et al., 2018); and a PFAM Glyoxalase bleomycin resistance protein dioxygenase (gene ID 2677), which is possibly involved in the antioxidant defense system of cyanobacteria, mediating detoxification of the abiotic stress response intermediate methylglyoxal (Rai et al., 2021; Suttisansanee & Honek, 2011).

Additionally, a NUDIX (**nu**cleoside **di**phosphates linked to **x**) hydrolase (gene ID 2479), with the ability to hydrolyze a wide range of pyrophosphates (Bessman et al., 1996; McLennan, 2006), was detected to be fourfold less abundant at 55°C. While some Nudix superfamily members, such as MutT from *E. coli*, possess the ability to hydrolyze oxidized and potentially mutagenic nucleotides (McLennan, 2006), functions can also revolve around the control of metabolic intermediate or signaling compound levels (Bessman et al., 1996).

**Figure 10:**
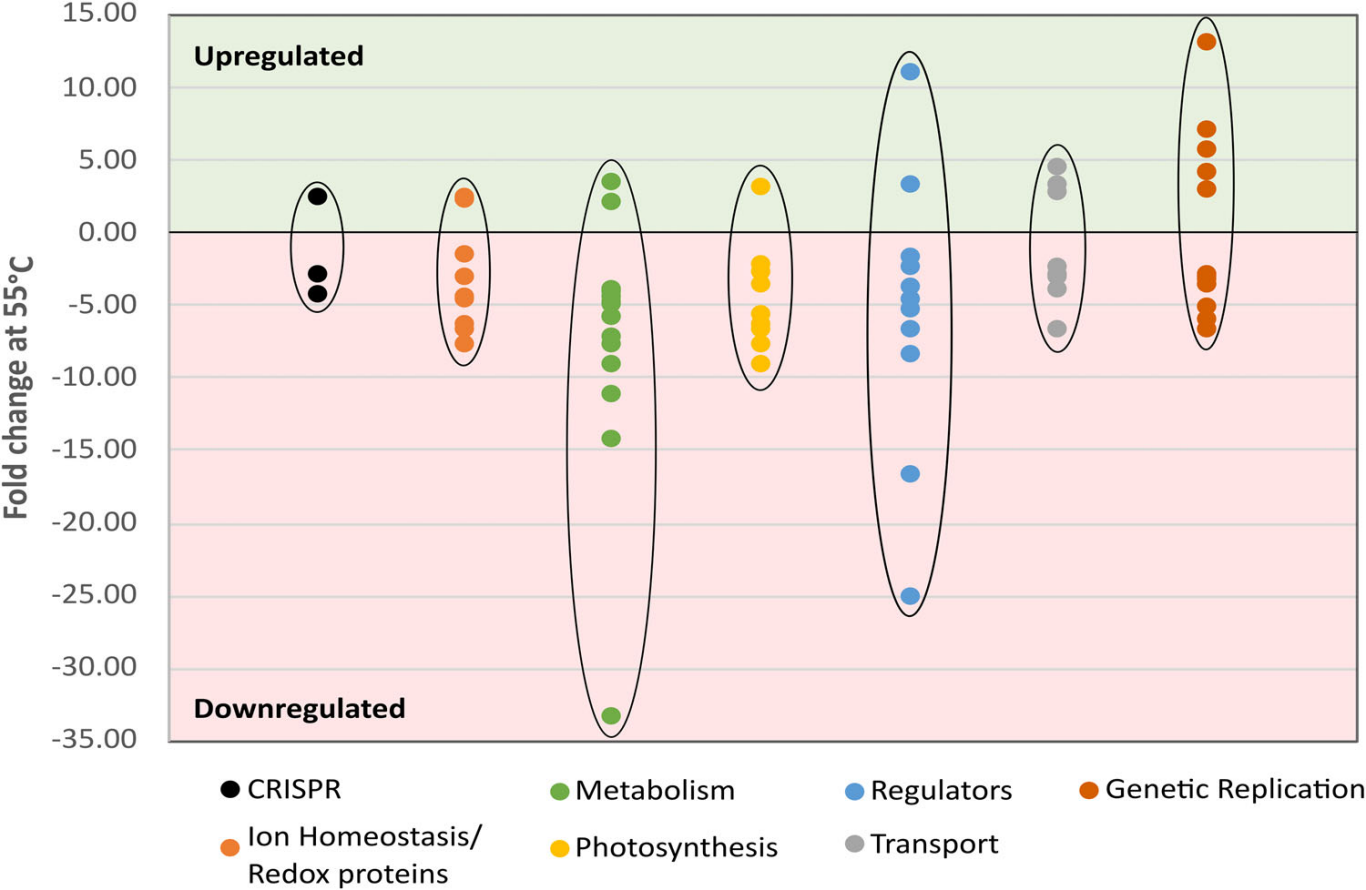
Selected differential protein expression levels of Thermosynechococcaceae cyanobacterium sp. Okahandja at 55°C in relation to 40°C. Proteins involved in ion homeostasis or redox chemistry, metabolism, photosynthesis, and regulation show tendencies toward downregulation, while CRISPR, transport, or genetic replication‐related proteins exhibit both increased and decreased expression levels.

Finally, a bifunctional pantoate‐beta‐alanine ligase/dCMP kinase (gene ID 312) was detected as approximately 3.9‐fold downregulated at 55°C throughout all samples. It represents a fusion of two enzymes that is unique to cyanobacteria according to the InterPro database (Blum et al., 2021; InterPro Entry IPR024894, 2024; Paysan‐Lafosse et al., 2023). First, the pantoate‐beta‐alanine ligase catalyzes the condensation of pantoate with beta‐alanine in an ATP‐dependent reaction via a pantoyl‐adenylate intermediate (Miyatake et al., 1979). Second, the dCMP kinase catalyzes the transfer of a phosphate group from ATP to either CMP or dCMP to form CDP or dCDP and ADP, respectively (Briozzo et al., 1998). Overall, apart from the phosphoribosylglycinamide formyltransferase, seemingly non‐essential pathways were downregulated, hazarding the consequences of a higher vulnerability toward radical oxygen species and less metabolic flexibility. Figure 10 provides an overview of the fold changes at 55°C in relation to protein expression levels at 40°C of all discussed proteins. Refer to Table A2 for a comprehensive list of these proteins or the supplementary material for all 129 differentially expressed proteins in table form: https://zenodo.org/doi/10.5281/zenodo.10007199.

##### Bridging the gap: Integrating the findings with established research

Expectantly, in most thermophiles' proteomes such as *Pyrococcus furiosus* (Shockley et al., 2003), *Thermotoga maritima* (Pysz et al., 2004), or *Thermoanaerobacter tengcongensis* (Wang et al., 2007), heat shock proteins (HSPs) were abundant at elevated temperatures, forming complexes with other proteins to transmit thermal protection.

Despite the heat‐inducible repressive HSP regulator HrcA (gene ID 2557) being strongly downregulated in the proteome of Thermosynechococcaceae cyanobacterium sp. Okahandja at 50°C and 55°C, thus theoretically enabling HSP upregulation, no actual heat shock protein fragments could be detected through MS/MS chromatography. Possible reasons for the lack of HSPs could be inadequate methodological sensitivity, incomplete protein extraction from cyanobacterial cells, or insufficiently high temperature during cultivation necessary for HSP induction in the investigated strain.

Consistent with the results from Chen et al. regarding the heat adaptations from *T. tengcongensis* (Chen et al., 2013), redox respiratory proteins such as ferredoxin, NADH‐quinone oxidoreductase (subunit J), and NAD(P)H‐quinone oxidoreductase (subunit C) were downregulated. Additionally, we found hints of (attempted) increased glucosamine recruitment through 4.5‐fold upregulation of the putative chitobiose permease, which would be in line with the observed shift toward carbohydrate utilization for augmentation of energy generation in *T. tengcongensis* (Chen et al., 2013; Wang et al., 2007).

However, we could gather no evidence to support glucosamine to fructose conversion, as seen in *T. tengcongensis* (Chen et al., 2013). In that context, the data did generally also not suggest an increased metabolic flow toward fructose through enhanced expression of phosphomannomutase or glucosamine‐6‐P deaminase. Quite the opposite, the fructose‐6‐P generating glucose‐6‐isomerase (gene ID 2040) was downregulated incrementally with increasing temperature. However, continuous upregulation of phosphorylation catalyzing fructosamine kinase (gene ID 917) might indicate metabolic flow toward fructosamines formed by glycation. Although the importance of glycolysis in the heat response was demonstrated in systems biology‐informed studies of several thermophiles including *Thermotoga maritima* (Wang et al., 2012), *Geobacillus thermoglucosidasius* (Loftie‐Eaton et al., 2013), or *Thermus thermophilus* (Trauger et al., 2008), no glycolysis‐related peptide fragments could be retrieved from our data set. Moreover, no further overlaps regarding upregulated proteins in response to elevated temperatures between Thermosynechococcaceae cyanobacterium sp. Okahandja and *T. tengcongensis* could be identified.

From the persistent downregulation of a chorismate mutase, also known as chorismate pyruvatemutase (gene ID 1170), and a citramalate synthase (gene ID 955), a potential lack of pyruvate could be deduced, as it is a substrate of both enzymes. In contrast to this observation, Chen et al. (2013) reported an increased flux toward pyruvate, mediated by glyceraldehyde‐3‐phosphate dehydrogenase. Furthermore, cistronic operon organization or gene clustering was reported in several other thermophilic organisms (Chen et al., 2013; Wang et al., 2015) but was not observed in our data set, where the differentially expressed proteins were rather scattered within the genome.

## 4 CONCLUSIONS

In this study, we present a novel thermophilic cyanobacterium species obtained from a hot spring near Okahandja, Namibia. PacBio‐guided whole‐genome sequencing of its genome and subsequent alignment with the closely related genome of *P. lividus* PCC 6715 revealed substantial gene flux events, putatively owed to local microbial community dynamics and ecological adaptations (Cadillo‐Quiroz et al., 2012; Miller & Carvey, 2019; Schmutzer & Barraclough, 2019). Morphologically, it resembles other *Thermosynechococcus* spp. with its solitary, cylindrical cells and polar granulates, as determined with STEM, LEI, and brightfield microscopy (Komárek et al., 2020). Functional annotation with BlastKOALA (KEGG Orthology And Links Annotation) confirmed the two required genes for glycogen biosynthesis, glucose‐1‐phosphate adenylyltransferase, and starch synthase in the genome, as known from other cyanobacteria (Suzuki et al., 2013). While the 16S ribosomal RNA gene sequence exhibited a sequence similarity of 98.87% with that of the strain *P. lividus* PCC 6715, a maximum likelihood‐based phylogenetic analysis (Figure 4) placed the isolate in a closely related, yet separate branch. Further indications for allopatric (geographically isolated) speciation were provided by a whole‐genome sequence‐based ANI of 81% between the two strains, with 95% representing the threshold for identical species (Ciufo et al., 2018).

Following the example of Jahodářová et al. (2022), we analyzed the CRISPR/Cas adaptive immune system and conducted ortholog‐inferred phylogenetics of our isolated strain with respect to *Parathermosynechococcus* spp. and Thermosynechococcaceae, respectively. While insights about the local phage predation pressures of the respective strains could be gained (Westra et al., 2016), the vast difference in sampling time difference renders it difficult to compare their data. As expected, considering the geological and temporal distance between the strain of this study (Okahandja, Namibia, 2024) and *P. lividus* PCC 6715 (Yellowstone National Park, USA, 1961), no shared CRISPR spacer sequences could be detected. Furthermore, probably owed to the limited accessibility of phage sequences (Andersson & Banfield, 2008; Westra et al., 2016), no matches with virus genome sequences were yielded with a nucleotide BLAST search of all CRISPR spacer sequences.

In addition to the phylogenetic analysis based on 16S rRNA gene sequences, the orthology‐based phylogenetic analysis (Figure 5) supported the notion that Thermosynechococcaceae cyanobacterium sp. Okahandja represents a novel genus within the family of Thermosynechococcaceae, which is closely related to the novel genus *Parathermosynechococcus* (Tang et al., 2024). Of its 2276 orthogroups, representing 93.8% of all genome‐encoded genes, the Namibian strain shared 2257 orthogroups with *P. lividus* PCC 6715, while differing in 78 genes or 2.9% of its genome.

Furthermore, the present study shed light on the proteomic adaptations to varying temperatures of the isolated cyanobacterium. By investigating the overexpression of several key proteins in response to elevated temperatures, we have gained valuable insights into the mechanisms underlying the thermotolerance of this cyanobacterium. One important area of exploration is the identification and characterization of the specific genes and regulatory mechanisms that govern the observed overexpression of specific proteins in response to elevated temperatures. Understanding the molecular basis of these adaptations will provide valuable knowledge about the strategies employed by Thermosynechococcaceae cyanobacterium sp. Okahandja and other thermophilic cyanobacteria to thrive in extreme thermal environments. Genetic engineering approaches, such as gene knockout or overexpression, may offer opportunities to enhance the thermotolerance of other organisms or even develop novel biotechnological applications. An especially interesting candidate for such studies could be the phytoene synthase that was revealed through biosynthetic gene cluster inquiry.

Additionally, it would be intriguing to explore the broader ecological significance of the observed proteomic adaptations of Thermosynechococcaceae cyanobacterium sp. Okahandja. Understanding how these adaptations affect the overall fitness and ecological success of this cyanobacterium in its natural habitat, such as hot springs, would provide a more comprehensive picture of its role in these ecosystems. In this study, the adaptation of protein expression patterns of a new Thermosynechococcaceae cyanobacterium species to varying temperatures has provided valuable insights into the mechanisms underlying its thermotolerance. This research sets the stage for future investigations aimed at deciphering the regulatory pathways for the attainment of thermotolerance in cyanobacteria.

## AUTHOR CONTRIBUTIONS

**Nathanael D. Arnold**: Conceptualization; methodology; software; validation; formal analysis; data curation; writing—original draft; writing—review and editing; visualization. **Michael Paper**: Conceptualization; methodology; software; validation; formal analysis; investigation; data curation; writing—original draft; writing—review and editing; visualization. **Tobias Fuchs**: Investigation. **Nadim Ahmad**: Investigation. **Patrick Jung**: Software; validation; visualization. **Michael Lakatos**: Resources; validation. **Katia Rodewald**: Methodology; investigation; visualization. **Bernhard Rieger**: Resources. **Farah Qoura**: Supervision; project administration. **Martha Kandawa‐Schulz**: Writing—review and editing. **Norbert Mehlmer**: Investigation; supervision. **Thomas B. Brüc**k: Conceptualization; methodology; resources; writing—review and editing; supervision; project administration; funding acquisition.

## CONFLICT OF INTEREST STATEMENT

None declared.

## ETHICS STATEMENT

None required.

## Data Availability

All relevant data are provided in full in the results and appendix section of this paper. Additional analyses and raw data sets can be accessed in the Zenodo repository at https://zenodo.org/doi/10.5281/zenodo.10007199. The Thermosynechococcaceae cyanobacterium sp. Okahandja genome can be found at NCBI under accession CP136945. The 16S rRNA gene sequence can be accessed at GenBank under OR689577 or in Appendix Table A1.

## 1

**Table A1:** Thermosynechococcaceae cyanobacterium sp. Okahandja full 16S ribosomal RNA gene sequence (extracted from the genome).

Thermosynechococcaceae cyanobacterium sp. Okahandja full 16S ribosomal RNA gene sequence (extracted from genome) ATGGAGAGTTTGATCCTGGCTCAGGATGAACGCTGGCGGTCTGCTTAACACATGCAAGTCGAACGGGCTCTTCGGAGCTAGTGGCGGACGGGTGAGTAACACGTGAGAATCTACCCTTAGGAGGGGGATAACAGTTGGAAACGACTGCTAATACCCCATATGCCGCAAGGTGAAATCTTTTTGGCCTGAGGATGAGCTCGCGGTGGATTAGCTAGTTGGTGGGGTAATGGCCTACCAAGGCAACGATCCATAGCTGGTCTGAGAGGATGATCAGCCACACTGGGACTGAGACACGGCCCAGACTCCTACGGGAGGCAGCAGTGGGGAATTTTCCGCAATGGGCGAAAGCCTGACGGAGCAAGACCGCGTGAGGGACGAAGGCCCTTGGGTTGTAAACCTCTTTTCTCAGGGAAGAACACAATGACGGTACCTGAGGAATAAGCCTCGGCTAACTCCGTGCCAGCAGCCGCGGTAAGACGGAGGAGGCAAGCGTTATCCGGAATTATTGGGCGTAAAGCGTCCGCAGGTGGCTTTCCAAGTCTGCTGTTAAAGCCCGAGGCTCAACCTCGGATCGGCGGTGGAAACTGGATCGCTAGAGTGCGTCAGGGGTAGGGGGAATTCCCGGTGTAGCGGTGAAATGCGTAGATATCGGGAAGAACACCAGCGACGAAAGTGCCCTACTGGGACGTTACTGACACTCATGGACGAAAGCTAGGGGAGCGAAAGGGATTAGATACCCCTGTAGTCCTAGCCGTAAACGATGGACACTAGGTGTTGCTGGTATCCACTCCAGCAGTGCCGTAGCCAACGCGTTAAGTGTCCCGCCTGGGGAGTACGCACGCAAGTGTGAAACTCAAAGGAATTGACGGGGGCCCGCACAAGCGGTGGAGTATGTGGTTTAATTCGATGCAACGCGAAGAACCTTACCAGGGCTTGACATGTCGCGAACCCCGCGGAAACGTGGGGGTGCCTTCGGGAGCGCGAACACAGGTGGTGCATGGCTGTCGTCAGCTCGTGTCGTGAGATGTTGGGTTAAGTCCCGCAACGAGCGCAACCCTCGTCCTTAGTTGCCAGCATTAAGTTGGGCACTCTAGGGAGACTGCCGGTGACAAACCGGAGGAAGGTGGGGATGACGTCAAGTCATCATGCCCCTTACGCCCTGGGCTACACACGTACTACAATGCTGTGGACAGAGAGTTGCCAACCCGCGAGGGCGAGCTAATCTCTTAAACCACGGCTCAGTTCAGATTGCAGGCTGCAACTCGCCTGCATGAAGGCGGAATCGCTAGTAATCGCAGGTCAGCATACTGCGGTGAATACGTTCCCGGGCCTTGTACACACCGCCCGTCACACCATGGGAGTTGGCCACGCCCGAAGTCGTTACTCCAACCCGTAAGGGAGGGGGACGCCTAAGGCAGGGCTGATGACTGGGGTGAAGTCGTAACAAGGTAGCCGTACCGGAAGGTGTGGCTGGATCACCTCCTTT

**Table A2:** All upregulated proteins of Thermosynechococcaceae cyanobacterium sp. Okahandja in dependence of temperature. Log2 fold changes (Log2 FC) refer to expression levels at 40°C. Annotations were performed by NCBI's Prokaryotic Genome Annotation Pipeline (Stolyar et al., 2014; Tang et al., 2018, 2024), which utilizes the TIGRFAM (Kumar et al., 2018) database and eggNOG‐mapper (Buchfink et al., 2014; Kelly & Maini, 2013), deploying the eggNOG 5.0 database.

Function	Accession	50°C log 2FC	55°C log2 FC	Description
CRISPR	1513	−2.33	−4.17	CRISPR‐associated endonuclease Cas1
CRISPR	1993	1.99	−2.78	Type I‐E CRISPR‐associated protein Cas7/Cse4/CasC
CRISPR	1516	1.12	2.46	Type III‐B CRISPR‐associated protein Cas10/Cmr2
Ion Homeostasis/Redox proteins	1603	−2.63	−7.69	Peroxiredoxin
Ion Homeostasis/Redox proteins	2641	−4.55	−6.67	2Fe‐2S iron‐sulfur cluster‐binding protein
Ion Homeostasis/Redox proteins	363	1.15	−6.25	NADH‐quinone oxidoreductase subunit J
Ion Homeostasis/Redox proteins	2402	−1.35	−4.55	Peptide‐methionine (S)‐S‐oxide reductase MsrA
Ion Homeostasis/Redox proteins	553	−2.33	−4.35	AarF/ABC1/UbiB kinase family protein
Ion Homeostasis/Redox proteins	1190	−2.56	−3.03	Ferredoxin
Ion Homeostasis/Redox proteins	2070	2.37	−1.43	Ferredoxin thioredoxin reductase variable alpha chain
Ion Homeostasis/Redox proteins	1860	1.09	2.27	SUMF1/EgtB/PvdO family nonheme iron enzyme
Ion Homeostasis/Redox proteins	93	1.09	2.34	2Fe‐2S iron‐sulfur cluster‐binding protein
Ion Homeostasis/Redox proteins	1055	−1.41	2.38	Ferrous iron transport protein A
Metabolism	2698	−8.33	−33.33	Squalene‐‐hopene cyclase
Metabolism	1725	−5.88	−14.29	TldD/PmbA family protein
Metabolism	2289	−1.92	−11.11	Phosphoribosylglycinamide formyltransferase
Metabolism	190	−6.25	−9.09	M23 family metallopeptidase
Metabolism	2040	−5.88	−7.69	Glucose‐6‐phosphate isomerase
Metabolism	955	−3.23	−7.14	Citramalate synthase
Metabolism	2427	−1.18	−5.88	Glycosyltransferase family 1 protein
Metabolism	1135	−5.00	−5.88	Haloacid dehalogenase superfamily, subfamily IA
Metabolism	2708	−2.38	−5.00	Glycosyltransferase
Metabolism	2533	−4.55	−4.76	Deoxyhypusine synthase
Metabolism	2677	−1.89	−4.35	PFAM Glyoxalase bleomycin resistance protein dioxygenase
Metabolism	1170	−3.33	−4.17	Chorismate mutase
Metabolism	2479	−1.67	−4.00	NUDIX hydrolase
Metabolism	312	−1.05	−3.85	Bifunctional pantoate‐‐beta‐alanine ligase/(d)CMP kinase
Metabolism	917	1.35	2.17	fructosamine kinase family protein
Metabolism	104	−1.49	3.54	GNAT family N‐acetyltransferase
Photosynthesis	1482	−4.17	−9.09	HEAT repeat domain‐containing protein
Photosynthesis	623	−8.33	−7.69	Protoporphyrinogen oxidase
Photosynthesis	2629	−4.76	−6.67	Apocytochrome f
Photosynthesis	694	−2.17	−6.25	Photosystem I reaction center subunit II
Photosynthesis	1748	−2.70	−5.56	Chlorophyll a‐b binding domain‐containing protein
Photosynthesis	1572	−2.04	−3.57	Photosystem II manganese‐stabilizing polypeptide
Photosynthesis	1413	−1.05	−2.63	Photosynthetic/respiratory NAD(P)H‐quinone oxidoreductase subunit C
Photosynthesis	697	−1.14	−2.22	Chlororespiratory reduction protein 7
Photosynthesis	1483	1.16	3.15	Phycobilisome rod‐core linker polypeptide
Regulators	652	−5.26	−25.00	YciI family protein
Regulators	1449	−4.76	−16.67	YbaB/EbfC family nucleoid‐associated protein
Regulators	302	−2.86	−8.33	ATP‐binding protein
Regulators	1885	−1.35	−6.67	GGDEF domain
Regulators	1377	−2.38	−5.26	Metalloregulator ArsR/SmtB family transcription factor
Regulators	2557	−3.03	−4.55	Heat‐inducible transcriptional repressor HrcA
Regulators	1647	−2.17	−3.70	LysR family transcriptional regulator
Regulators	1886	−2.22	−2.33	Circadian clock protein KaiA
Regulators	2313	−3.85	−1.69	Translation elongation factor 4
Regulators	1693	1.28	3.22	Response regulator
Regulators	960	1.79	11.01	Signal Transduction Histidine Kinase
Transport	1624	−4.00	−6.67	Sulfate ABC transporter permease subunit CysT
Transport	2077	−2.94	−4.00	Energy‐coupling factor ABC transporter permease
Transport	710	−2.08	−3.03	Protein translocase subunit SecF
Transport	506	−1.14	−2.86	LPS export ABC transporter ATP‐binding protein
Transport	908	−1.14	−2.38	DUF4040 domain‐containing protein
Transport	1681	1.7	2.79	SLC13 family permease
Transport	1171	1	3.23	Signal peptide peptidase SppA
Transport	100	2.54	4.58	Carbohydrate ABC transporter permease
Genetic replication	2324	−1.82	−6.67	DNA adenine methylase
Genetic replication	906	−4.00	−5.88	Segregation/condensation protein A
Genetic replication	158	−2.44	−5.00	30S ribosomal protein S12
Genetic replication	418	1.11	−3.45	Pentapeptide repeat‐containing protein
Genetic replication	594	−2.44	−3.45	Polyribonucleotide nucleotidyltransferase
Genetic replication	2513	−2.38	−3.13	DNA‐3‐methyladenine glycosylase
Genetic replication	2297	−3.33	−2.94	Ribosome‐associated translation inhibitor RaiA
Genetic replication	2574	−2.70	−2.86	Ribonuclease H‐like domain‐containing protein
Genetic replication	2711	1.53	−2.78	Hypothetical protein pgaptmp_002711
Genetic replication	1611	1.16	3.02	DNA polymerase III subunit delta'
Genetic replication	421	1.13	4.3	Phenylalanine‐‐tRNA ligase subunit beta
Genetic replication	2046	2.72	5.79	Crossover junction endodeoxyribonuclease RuvC
Genetic replication	2673	1.23	7.13	50S ribosomal protein L33
Genetic replication	2469	3.03	13.13	Hypothetical protein pgaptmp_002469

Upregulated at:  55°C only  50°C and 55°C,  50°C only

Downregulated at:  55°C only,  50°C and 55°C increasingly,  50°C more, 55° C less again.

## References

[mbo370000-bib-0001] Abed, R. M. M. , Dobretsov, S. , & Sudesh, K. (2009). Applications of Cyanobacteria in biotechnology. Journal of Applied Microbiology, 106(1), 1–12. 19191979 10.1111/j.1365-2672.2008.03918.x

[mbo370000-bib-0002] Adomako, M. , Ernst, D. , Simkovsky, R. , Chao, Y.‐Y. , Wang, J. , Fang, M. , Bouchier, C. , Lopez‐Igual, R. , Mazel, D. , Gugger, M. , & Golden, SS (2022). Comparative genomics of *Synechococcus elongatus* explains the phenotypic diversity of the strains. mBio, 13(3), e0086222. 35475644 10.1128/mbio.00862-22 PMC9239245

[mbo370000-bib-0003] Afgan, E. , Baker, D. , van den Beek, M. , Blankenberg, D. , Bouvier, D. , Čech, M. , Chilton, J. , Clements, D. , Coraor, N. , Eberhard, C. , Grüning, B. , Guerler, A. , Hillman‐Jackson, J. , Von Kuster, G. , Rasche, E. , Soranzo, N. , Turaga, N. , Taylor, J. , Nekrutenko, A. , & Goecks, J. (2016). The Galaxy platform for accessible, reproducible and collaborative biomedical analyses: 2016 update. Nucleic Acids Research, 44(W1), W3–W10. 27137889 10.1093/nar/gkw343 PMC4987906

[mbo370000-bib-0004] Aimi, J. , Qiu, H. , Williams, J. , Zalkin, H. , & Dixon, J. E. (1990). De novo purine nucleotide biosynthesis: Cloning of human and avian cDNAs encoding the trifunctional glycinamide ribonucleotide synthetase‐aminoimidazole ribonucleotide synthetase‐glycinamide ribonucleotide transformylase by functional complementation in *E. coli* . Nucleic Acids Research, 18(22), 6665–6672. 2147474 10.1093/nar/18.22.6665 PMC332626

[mbo370000-bib-0005] Aksu, Z. , Ertuğrul, S. , & Dönmez, G. (2009). Single and binary chromium(VI) and Remazol Black B biosorption properties of *Phormidium* sp. Journal of Hazardous Materials, 168(1), 310–318. 19278783 10.1016/j.jhazmat.2009.02.027

[mbo370000-bib-0006] Allewalt, J. P. , Bateson, M. M. , Revsbech, N. P. , Slack, K. , & Ward, D. M. (2006). Effect of temperature and light on growth of and photosynthesis by *Synechococcus* isolates typical of those predominating in the octopus spring microbial mat community of Yellowstone National Park. Applied and Environmental Microbiology, 72(1), 544–550. 16391090 10.1128/AEM.72.1.544-550.2006 PMC1352173

[mbo370000-bib-0007] Anderson, R. E. , Brazelton, W. J. , & Baross, J. A. (2011). Using CRISPRs as a metagenomic tool to identify microbial hosts of a diffuse flow hydrothermal vent viral assemblage. FEMS Microbiology Ecology, 77(1), 120–133. 21410492 10.1111/j.1574-6941.2011.01090.x

[mbo370000-bib-0008] Andersson, A. F. , & Banfield, J. F. (2008). Virus population dynamics and acquired virus resistance in natural microbial communities. Science, 320(5879), 1047–1050. 18497291 10.1126/science.1157358

[mbo370000-bib-0009] Anitori, R. P. , Trott, C. , Saul, D. J. , Bergquist, P. L. , & Walter, M. R. (2002). A culture‐independent survey of the bacterial community in a radon hot spring. Astrobiology, 2(3), 255–270. 12530236 10.1089/153110702762027844

[mbo370000-bib-0010] Arndt, D. , Grant, J. R. , Marcu, A. , Sajed, T. , Pon, A. , Liang, Y. , & Wishart, D. S. (2016). PHASTER: A better, faster version of the PHAST phage search tool. Nucleic Acids Research, 44(W1), W16–W21. 27141966 10.1093/nar/gkw387 PMC4987931

[mbo370000-bib-0011] Auch, A. F. , Klenk, H.‐P. , & Göker, M. (2010). Standard operating procedure for calculating genome‐to‐genome distances based on high‐scoring segment pairs. Standards in Genomic Sciences, 2(1), 142–148. 21304686 10.4056/sigs.541628 PMC3035261

[mbo370000-bib-0012] Aziz, R. K. , Bartels, D. , Best, A. A. , DeJongh, M. , Disz, T. , Edwards, R. A. , Formsma, K. , Gerdes, S. , Glass, E. M. , Kubal, M. , Meyer, F. , Olsen, G. J. , Olson, R. , Osterman, A. L. , Overbeek, R. A. , McNeil, L. K. , Paarmann, D. , Paczian, T. , Parrello, B. , … Zagnitko, O. (2008). The RAST server: Rapid annotations using subsystems technology. BMC Genomics, 9(1), 75. 18261238 10.1186/1471-2164-9-75 PMC2265698

[mbo370000-bib-0013] Barrangou, R. , Fremaux, C. , Deveau, H. , Richards, M. , Boyaval, P. , Moineau, S. , Romero, D. A. , & Horvath, P. (2007). CRISPR provides acquired resistance against viruses in prokaryotes. Science, 315(5819), 1709–1712. 17379808 10.1126/science.1138140

[mbo370000-bib-0014] Basak, S. , Banerjee, T. , Gupta, S. K. , & Ghosh, T. C. (2004). Investigation on the causes of codon and amino acid usages variation between thermophilic *Aquifex aeolicus* and mesophilic *Bacillus subtilis* . Journal of Biomolecular Structure and Dynamics, 22(2), 205–214. 15317481 10.1080/07391102.2004.10506996

[mbo370000-bib-0015] Berg Miller, M. E. , Yeoman, C. J. , Chia, N. , Tringe, S. G. , Angly, F. E. , Edwards, R. A. , Flint, H. J. , Lamed, R. , Bayer, E. A. , & White, B. A. (2012). Phage–bacteria relationships and CRISPR elements revealed by a metagenomic survey of the rumen microbiome. Environmental Microbiology, 14(1), 207–227. 22004549 10.1111/j.1462-2920.2011.02593.x

[mbo370000-bib-0016] Bessman, M. J. , Frick, D. N. , & O'Handley, S. F. (1996). The MutT proteins or “Nudix” hydrolases, a family of versatile, widely distributed, “housecleaning” enzymes. Journal of Biological Chemistry, 271(41), 25059–25062. 8810257 10.1074/jbc.271.41.25059

[mbo370000-bib-0017] Biehler, E. , Alkerwi, A. , Hoffmann, L. , Krause, E. , Guillaume, M. , Lair, M. L. , & Bohn, T. (2012). Contribution of violaxanthin, neoxanthin, phytoene and phytofluene to total carotenoid intake: Assessment in Luxembourg. Journal of Food Composition and Analysis, 25(1), 56–65.

[mbo370000-bib-0018] Blin, K. , Shaw, S. , Augustijn, H. E. , Reitz, Z. L. , Biermann, F. , Alanjary, M. , Fetter, A. , Terlouw, B. R. , Metcalf, W. W. , Helfrich, E. J. N. , van Wezel, G. P. , Medema, M. H. , Weber, T. (2023). antiSMASH 7.0: New and improved predictions for detection, regulation, chemical structures and visualisation. Nucleic Acids Research, 51, gkad344. 10.1093/nar/gkad344 PMC10320115 37140036

[mbo370000-bib-0019] Blum, M. , Chang, H.‐Y. , Chuguransky, S. , Grego, T. , Kandasaamy, S. , Mitchell, A. , Nuka, G. , Paysan‐Lafosse, T. , Qureshi, M. , Raj, S. , Richardson, L. , Salazar, G. A. , Williams, L. , Bork, P. , Bridge, A. , Gough, J. , Haft, D. H. , Letunic, I. , Marchler‐Bauer, A. , … Finn, R. D. (2021). The InterPro protein families and domains database: 20 years on. Nucleic Acids Research, 49(D1), D344–D354. 33156333 10.1093/nar/gkaa977 PMC7778928

[mbo370000-bib-0020] Brettin, T. , Davis, J. J. , Disz, T. , Edwards, R. A. , Gerdes, S. , Olsen, G. J. , Olson, R. , Overbeek, R. , Parrello, B. , Pusch, G. D. , Shukla, M. , Thomason, J. A. , Stevens, R. , Vonstein, V. , Wattam, A. R. , & Xia, F. (2015). RASTtk: A modular and extensible implementation of the RAST algorithm for building custom annotation pipelines and annotating batches of genomes. Scientific Reports, 5(1), 8365. 25666585 10.1038/srep08365 PMC4322359

[mbo370000-bib-0021] Briozzo, P. , Golinelli‐Pimpaneau, B. , Gilles, A. M. , Gaucher, J. F. , Burlacu‐Miron, S. , Sakamoto, H. , Janin, J. , & Bârzu, O. (1998). Structures of *Escherichia coli* CMP kinase alone and in complex with CDP: A new fold of the nucleoside monophosphate binding domain and insights into cytosine nucleotide specificity. Structure, 6(12), 1517–1527. 9862805 10.1016/s0969-2126(98)00150-6

[mbo370000-bib-0022] Browning, D. F. , & Busby, S. J. W. (2004). The regulation of bacterial transcription initiation. Nature Reviews Microbiology, 2(1), 57–65. 15035009 10.1038/nrmicro787

[mbo370000-bib-0023] Buchfink, B. , Xie, C. , & Huson, D. H. (2014). Fast and sensitive protein alignment using DIAMOND. Nature Methods, 12(1), 59–60. 25402007 10.1038/nmeth.3176

[mbo370000-bib-0024] Bunker, R. D. , McKenzie, J. L. , Baker, E. N. , & Arcus, V. L. (2008). Crystal structure of PAE0151 from *Pyrobaculum aerophilum*, a PIN‐domain (VapC) protein from a toxin‐antitoxin operon. Proteins: Structure, Function, and Bioinformatics, Jul 1 72(1), 510–518. 10.1002/prot.22048 18398909

[mbo370000-bib-0025] Burnat, M. , Li, B. , Kim, S. H. , Michael, A. J. , & Flores, E. (2018). Homospermidine biosynthesis in the cyanobacterium *Anabaena* requires a deoxyhypusine synthase homologue and is essential for normal diazotrophic growth. Molecular Microbiology, 109(6), 763–780. 29923645 10.1111/mmi.14006

[mbo370000-bib-0026] Burra, P. V. , Kalmar, L. , & Tompa, P. (2010). Reduction in structural disorder and functional complexity in the thermal adaptation of prokaryotes. PLoS One, 5(8), e12069. 20711457 10.1371/journal.pone.0012069 PMC2920320

[mbo370000-bib-0027] Bywaters, K. F. , & Fritsen, C. H. (2015). Biomass and neutral lipid production in geothermal microalgal consortia. Frontiers in Bioengineering and Biotechnology, 2, 82. 25763368 10.3389/fbioe.2014.00082 PMC4329875

[mbo370000-bib-0028] Cadillo‐Quiroz, H. , Didelot, X. , Held, N. L. , Herrera, A. , Darling, A. , Reno, M. L. , Krause, D. J. , & Whitaker, R. J. (2012). Patterns of gene flow define species of thermophilic Archaea. PLoS Biology, 10(2), e1001265. 22363207 10.1371/journal.pbio.1001265 PMC3283564

[mbo370000-bib-0029] Callieri, C. , Slabakova, V. , Dzhembekova, N. , Slabakova, N. , Peneva, E. , Cabello‐Yeves, P. J. , Di Cesare, A. , Eckert, E. M. , Bertoni, R. , Corno, G. , Salcher, M. M. , Kamburska, L. , Bertoni, F. , & Moncheva, S. (2019). The mesopelagic anoxic Black Sea as an unexpected habitat for *Synechococcus* challenges our understanding of global “deep red fluorescence”. The ISME Journal, 13(7), 1676–1687. 30820035 10.1038/s41396-019-0378-z PMC6776005

[mbo370000-bib-0030] Camacho, C. , Coulouris, G. , Avagyan, V. , Ma, N. , Papadopoulos, J. , Bealer, K. , & Madden, T. L. (2009). BLAST+: Architecture and applications. BMC Bioinformatics, 10(1), 421. 20003500 10.1186/1471-2105-10-421 PMC2803857

[mbo370000-bib-0031] Chen, Z. , Wen, B. , Wang, Q. , Tong, W. , Guo, J. , Bai, X. , Zhao, J. , Sun, Y. , Tang, Q. , Lin, Z. , Lin, L. , & Liu, S. (2013). Quantitative proteomics reveals the temperature‐dependent proteins encoded by a series of cluster genes in *Thermoanaerobacter Tengcongensis* . Molecular & Cellular Proteomics, 12(8), 2266–2277. 23665590 10.1074/mcp.M112.025817 PMC3734584

[mbo370000-bib-0032] Ciufo, S. , Kannan, S. , Sharma, S. , Badretdin, A. , Clark, K. , Turner, S. , Brover, S. , Schoch, C. L. , Kimchi, A. , & DiCuccio, M. (2018). Using average nucleotide identity to improve taxonomic assignments in prokaryotic genomes at the NCBI. International Journal of Systematic and Evolutionary Microbiology, 68(7), 2386–2392. 29792589 10.1099/ijsem.0.002809 PMC6978984

[mbo370000-bib-0033] Cooper, C. R. , Daugherty, A. J. , Tachdjian, S. , Blum, P. H. , & Kelly, R. M. (2009). Role of vapBC toxin–antitoxin loci in the thermal stress response of *Sulfolobus solfataricus* . Biochemical Society Transactions, 37(Pt 1), 123–126. 19143615 10.1042/BST0370123 PMC2919284

[mbo370000-bib-0034] Copeland, J. J. (1936). Yellowstone thermal myxophyceae. Annals of the New York Academy of Sciences, 36(1), 4–223.

[mbo370000-bib-0035] Darling, A. E. , Mau, B. , & Perna, N. T. (2010). Progressivemauve: Multiple genome alignment with gene gain, loss and rearrangement. PLoS One, 5(6), e11147. 20593022 10.1371/journal.pone.0011147 PMC2892488

[mbo370000-bib-0036] Demirci, H. , Wang, L. , Murphy, F. V. , Murphy, E. L. , Carr, J. F. , Blanchard, S. C. , Jogl, G. , Dahlberg, A. E. , & Gregory, S. T. (2013). The central role of protein S12 in organizing the structure of the decoding site of the ribosome. RNA, 19(12), 1791–1801. 24152548 10.1261/rna.040030.113 PMC3884664

[mbo370000-bib-0037] Dyer, D. L. , & Gafford, R. D. (1961). Some characteristics of a thermophilic blue‐green alga. Science, 134(3479), 616–617. 13725365 10.1126/science.134.3479.616

[mbo370000-bib-0038] Emerson, J. B. , Andrade, K. , Thomas, B. C. , Norman, A. , Allen, E. E. , Heidelberg, K. B. , & Banfield, J. F. (2013). Virus‐host and CRISPR dynamics in archaea‐dominated hypersaline Lake tyrrell, Victoria, Australia. Archaea, 2013, 1–12. 10.1155/2013/370871 PMC3703381 23853523

[mbo370000-bib-0039] Emms, D. M. , & Kelly, S. (2015). OrthoFinder: Solving fundamental biases in whole genome comparisons dramatically improves orthogroup inference accuracy. Genome Biology, 16(1), 157. 26243257 10.1186/s13059-015-0721-2 PMC4531804

[mbo370000-bib-0040] Emms, D. M. , & Kelly, S. (2017). STRIDE: Species tree root inference from gene duplication events. Molecular Biology and Evolution, 34(12), 3267–3278. 29029342 10.1093/molbev/msx259 PMC5850722

[mbo370000-bib-0041] Emms, D. M. , & Kelly, S. (2018). STAG: Species tree inference from all genes. bioRxiv, 267914.

[mbo370000-bib-0042] Emms, D. M. , & Kelly, S. (2019). OrthoFinder: Phylogenetic orthology inference for comparative genomics. Genome Biology, 20(1), 238. 31727128 10.1186/s13059-019-1832-y PMC6857279

[mbo370000-bib-0043] Erdmann, S. , & Garrett, R. A. (2012). Selective and hyperactive uptake of foreign DNA by adaptive immune systems of an archaeon via two distinct mechanisms. Molecular Microbiology, 85(6), 1044–1056. 22834906 10.1111/j.1365-2958.2012.08171.x PMC3468723

[mbo370000-bib-0044] Ertuğrul, S. , Bakır, M. , & Dönmez, G. (2008). Treatment of dye‐rich wastewater by an immobilized thermophilic cyanobacterial strain: *Phormidium* sp. Ecological Engineering, 32(3), 244–248.

[mbo370000-bib-0045] Fish, S. A. , & Codd, G. A. (1994). Bioactive compound production by thermophilic and thermotolerant cyanobacteria (blue‐green algae). World Journal of Microbiology & Biotechnology, 10(3), 338–341. 24421023 10.1007/BF00414875

[mbo370000-bib-0046] Fuchs, T. , Melcher, F. , Rerop, Z. S. , Lorenzen, J. , Shaigani, P. , Awad, D. , Haack, M. , Prem, S. A. , Masri, M. , Mehlmer, N. , & Brueck, T. B. (2021). Identifying carbohydrate‐active enzymes of *Cutaneotrichosporon oleaginosus* using systems biology. Microbial Cell Factories, 20(1), 205. 34711240 10.1186/s12934-021-01692-2 PMC8555327

[mbo370000-bib-0047] Galtier, N. , & Lobry, J. R. (1997). Relationships between genomic G+C content, RNA secondary structures, and optimal growth temperature in prokaryotes. Journal of Molecular Evolution, 44(6), 632–636. 9169555 10.1007/pl00006186

[mbo370000-bib-0048] Gao, H. , Wang, Y. , Liu, X. , Yan, T. , Wu, L. , Alm, E. , Arkin, A. , Thompson, D. K. , & Zhou, J. (2004). Global transcriptome analysis of the heat shock response of *Shewanella oneidensis* . Journal of Bacteriology, 186(22), 7796–7803. 15516594 10.1128/JB.186.22.7796-7803.2004 PMC524878

[mbo370000-bib-0049] Gemayel, R. , Fortpied, J. , Rzem, R. , Vertommen, D. , Veiga‐da‐Cunha, M. , & Van Schaftingen, E. (2007). Many fructosamine 3‐kinase homologues in bacteria are ribulosamine/erythrulosamine 3‐kinases potentially involved in protein deglycation. The FEBS Journal, 274(17), 4360–4374. 17681011 10.1111/j.1742-4658.2007.05948.x

[mbo370000-bib-0050] Gerdes, K. , Christensen, S. K. , & Løbner‐Olesen, A. (2005). Prokaryotic toxin‐antitoxin stress response loci. Nature Reviews Microbiology, 3(5), 371–382. 15864262 10.1038/nrmicro1147

[mbo370000-bib-0051] Glauser, M. , Stirewalt, V. L. , Bryant, D. A. , Sidler, W. , & Zuber, H. (1992). Structure of the genes encoding the rod‐core linker polypeptides of *Mastigocladus laminusus* phycobilisomes and functional aspects of the phycobiliprotein/linker‐polypeptide interactions. European Journal of Biochemistry, 205(3), 927–937. 1577010 10.1111/j.1432-1033.1992.tb16859.x

[mbo370000-bib-0052] Gophna, U. , Kristensen, D. M. , Wolf, Y. I. , Popa, O. , Drevet, C. , & Koonin, E. V. (2015). No evidence of inhibition of horizontal gene transfer by CRISPR–Cas on evolutionary timescales. The ISME Journal: Multidisciplinary Journal of Microbial Ecology, 9(9), 2021–2027. 10.1038/ismej.2015.20 PMC4542034 25710183

[mbo370000-bib-0053] Graverholt, O. S. , & Eriksen, N. T. (2007). Heterotrophic high‐cell‐density fed‐batch and continuous‐flow cultures of *Galdieria sulphuraria* and production of phycocyanin. Applied Microbiology and Biotechnology, 77(1), 69–75. 17786429 10.1007/s00253-007-1150-2

[mbo370000-bib-0054] Haft, D. H. (2001). TIGRFAMs: A protein family resource for the functional identification of proteins. Nucleic Acids Research, 29(1), 41–43. 11125044 10.1093/nar/29.1.41 PMC29844

[mbo370000-bib-0055] Haft, D. H. (2003). The TIGRFAMs database of protein families. Nucleic Acids Research, 31(1), 371–373. 12520025 10.1093/nar/gkg128 PMC165575

[mbo370000-bib-0056] Haft, D. H. , Selengut, J. D. , Richter, R. A. , Harkins, D. , Basu, M. K. , & Beck, E. (2013). TIGRFAMs and genome properties in 2013. Nucleic Acids Research, 41(Database issue), 387–395. 10.1093/nar/gks1234 PMC3531188 23197656

[mbo370000-bib-0057] Haft, D. H. , DiCuccio, M. , Badretdin, A. , Brover, V. , Chetvernin, V. , O'Neill, K. , Li, W. , Chitsaz, F. , Derbyshire, M. K. , Gonzales, N. R. , Gwadz, M. , Lu, F. , Marchler, G. H. , Song, J. S. , Thanki, N. , Yamashita, R. A. , Zheng, C. , Thibaud‐Nissen, F. , Geer, L. Y. , … Pruitt, K. D. (2018). RefSeq: An update on prokaryotic genome annotation and curation. Nucleic Acids Research, 46(D1), D851–D860. 29112715 10.1093/nar/gkx1068 PMC5753331

[mbo370000-bib-0058] Hansen, T. , Oehlmann, M. , & Schönheit, P. (2001). Novel type of glucose‐6‐phosphate someraseisomerase in the hyperthermophilic *Archaeon Pyrococcus* furiosus. Journal of Bacteriology, 183(11), 3428–3435. 11344151 10.1128/JB.183.11.3428-3435.2001 PMC99641

[mbo370000-bib-0059] Hitch, T. C. A. , Riedel, T. , Oren, A. , Overmann, J. , Lawley, T. D. , & Clavel, T. (2021). Automated analysis of genomic sequences facilitates high‐throughput and comprehensive description of bacteria. ISME Communications, 1(1), 16. 36732617 10.1038/s43705-021-00017-z PMC9723785

[mbo370000-bib-0060] Hongmei, J. , Aitchison, J. C. , Lacap, D. C. , Peerapornpisal, Y. , Sompong, U. , & Pointing, S. B. (2005). Community phylogenetic analysis of moderately thermophilic cyanobacterial mats from China, the Philippines and Thailand. Extremophiles, 9(4), 325–332. 15970994 10.1007/s00792-005-0456-1

[mbo370000-bib-0061] Hoshino, T. , Nakano, S. I. , Kondo, T. , Sato, T. , & Miyoshi, A. (2004). Squalene–hopene cyclase: Final deprotonation reaction, conformational analysis for the cyclization of (3R,S)−2,3‐oxidosqualene and further evidence for the requirement of an isopropylidene moiety both for initiation of the polycyclization cascade and for the formation of the 5‐membered E‐ring. Organic and Biomolecular Chemistry, 2(10), 1456–1470. 15136801 10.1039/b401172d

[mbo370000-bib-0062] Howell, D. M. , Xu, H. , & White, R. H. (1999). (R)‐citramalate synthase in methanogenic archaea. Journal of Bacteriology, 181(1), 331–333. 9864346 10.1128/jb.181.1.331-333.1999 PMC103565

[mbo370000-bib-0063] Hsueh, H. T. , Chu, H. , & Chang, C. C. (2007). Identification and characteristics of a cyanobacterium isolated from a hot spring with dissolved inorganic carbon. Environmental Science & Technology, 41(6), 1909–1914. 17410783 10.1021/es0620639

[mbo370000-bib-0064] Huerta‐Cepas, J. , Forslund, K. , Coelho, L. P. , Szklarczyk, D. , Jensen, L. J. , von Mering, C. , & Bork, P. (2017). Fast genome‐wide functional annotation through orthology assignment by eggNOG‐Mapper. Molecular Biology and Evolution, 34(8), 2115–2122. 28460117 10.1093/molbev/msx148 PMC5850834

[mbo370000-bib-0065] Huerta‐Cepas, J. , Szklarczyk, D. , Heller, D. , Hernández‐Plaza, A. , Forslund, S. K. , Cook, H. , Mende, D. R. , Letunic, I. , Rattei, T. , Jensen, L. J. , von Mering, C. , & Bork, P. (2019). eggNOG 5.0: A hierarchical, functionally and phylogenetically annotated orthology resource based on 5090 organisms and 2502 viruses. Nucleic Acids Research, 47(D1), D309–D314. 30418610 10.1093/nar/gky1085 PMC6324079

[mbo370000-bib-0066] Hur, S. , & Bruice, T. C. (2003). From the Cover: The near attack conformation approach to the study of the chorismate to prephenate reaction. Proceedings of the National Academy of Sciences USA, 100(21), 12015–12020. 10.1073/pnas.1534873100 PMC218705 14523243

[mbo370000-bib-0067] InterPro Entry IPR024894. (2024). Bifunctional pantoate ligase/cytidylate kinase [Internet]. https://www.ebi.ac.uk/interpro/entry/InterPro/IPR024894/

[mbo370000-bib-0068] Ionescu, D. , Hindiyeh, M. , Malkawi, H. , & Oren, A. (2010). Biogeography of thermophilic Cyanobacteria: Insights from the Zerka Ma'in hot springs (Jordan). FEMS Microbiology Ecology, 72(1), 103–113. 20180851 10.1111/j.1574-6941.2010.00835.x

[mbo370000-bib-0069] Jahodářová, E. , Poulíčková, A. , & Dvořák, P. (2022). The CRISPR/Cas machinery evolution and gene flow in the hot spring cyanobacterium *Thermostichus* . Diversity, 14(7), 502.

[mbo370000-bib-0070] Jain, C. , Rodriguez‐R, L. M. , Phillippy, A. M. , Konstantinidis, K. T. , & Aluru, S. (2018). High throughput ANI analysis of 90K prokaryotic genomes reveals clear species boundaries. Nature Communications, 9(1), 5114. 10.1038/s41467-018-07641-9 PMC6269478 30504855

[mbo370000-bib-0071] Jarett, J. , Dunfield, P. , Peura, S. , Wielen, P. , Hedlund, B. , Elshahed, M. , Kormas, K. , Teske, A. , Stott, M. , Birkeland, N.‐K. , Zhang, C. , Rengefors, K. , Lindemann, S. , Ravin, N. V. , Spear, J. , Hallam, S. , Crowe, S. , Steele, J. , Goudeau, D. , … Woyke, J. (2014). *Microbial dark matter phase II: Stepping deeper into unknown territory* (LBNL Report No. LBNL‐7076E). Lawrence Berkeley National Laboratory. Retrieved from https://escholarship.org/uc/item/3fs016dg

[mbo370000-bib-0072] Jeruzalmi, D. , Yurieva, O. , Zhao, Y. , Young, M. , Stewart, J. , Hingorani, M. , O'Donnell, M. , & Kuriyan, J. (2001). Mechanism of processivity clamp opening by the delta subunit wrench of the clamp loader complex of *E. coli* DNA polymerase III. Cell, 106(4), 417–428. 11525728

[mbo370000-bib-0073] Jung, P. , Briegel‐Williams, L. , Werner, L. , Jost, E. , Schultz, M. , Nürnberg, D. J. , Grube, M. , & Lakatos, M. (2024). A direct PCR approach with low‐biomass insert opens new horizons for molecular sciences on cryptogam communities. Applied and Environmental Microbiology, 90(3), e0002424. 38349146 10.1128/aem.00024-24 PMC10952543

[mbo370000-bib-0074] Kamruzzaman, M. , & Iredell, J. (2019). A ParDE‐family toxin antitoxin system in major resistance plasmids of *Enterobacteriaceae* confers antibiotic and heat tolerance. Scientific Reports, 9(1), 9872. 31285520 10.1038/s41598-019-46318-1 PMC6614396

[mbo370000-bib-0075] Kamruzzaman, M. , Wu, A. Y. , & Iredell, J. R. (2021). Biological functions of type II toxin‐antitoxin systems in bacteria. Microorganisms, 9(6), 1276. 34208120 10.3390/microorganisms9061276 PMC8230891

[mbo370000-bib-0076] Kanehisa, M. , Sato, Y. , & Morishima, K. (2016). BlastKOALA and GhostKOALA: KEGG tools for functional characterization of genome and metagenome sequences. Journal of Molecular Biology, 428(4), 726–731. 26585406 10.1016/j.jmb.2015.11.006

[mbo370000-bib-0077] Karatay, S. E. , & Dönmez, G. (2011). Microbial oil production from thermophile Cyanobacteria for biodiesel production. Applied Energy, 88(11), 3632–3635.

[mbo370000-bib-0078] Kelly, S. , & Maini, P. K. (2013). DendroBLAST: approximate phylogenetic trees in the absence of multiple sequence alignments. PLoS One, 8(3), e58537. 23554899 10.1371/journal.pone.0058537 PMC3598851

[mbo370000-bib-0079] Kleppe, K. , Ohtsuka, E. , Kleppe, R. , Molineux, I. , & Khorana, H. G. (1971). Studies on polynucleotides. Journal of Molecular Biology, 56(2), 341–361. 4927950 10.1016/0022-2836(71)90469-4

[mbo370000-bib-0080] Knoll, A. (2008). Cyanobacteria and Earth History. In: A. Herrero & E. Flores (Eds.), The Cyanobacteria: Molecular Biology, Genomics and Evolution. Caister Academic Press.

[mbo370000-bib-0081] Komárek, J. , Johansen, J. R. , Šmarda, J. , & Strunecký, O. (2020). Phylogeny and taxonomy of *Synechococcus*–like Cyanobacteria. Fottea, 20(2), 171–191.

[mbo370000-bib-0082] Koonin, E. V. , & Wolf, Y. I. (2009). Is evolution Darwinian or/and Lamarckian? Biology Direct, 4(1), 42. 19906303 10.1186/1745-6150-4-42 PMC2781790

[mbo370000-bib-0083] Koren, S. , Walenz, B. P. , Berlin, K. , Miller, J. R. , Bergman, N. H. , & Phillippy, A. M. (2017). Canu: Scalable and accurate long‐read assembly via adaptivek‐mer weighting and repeat separation. Genome Research, 27(5), 722–736. 28298431 10.1101/gr.215087.116 PMC5411767

[mbo370000-bib-0084] Krzywinski, M. , Schein, J. , Birol, I. , Connors, J. , Gascoyne, R. , Horsman, D. , Jones, S. J. , & Marra, M. A. (2009). Circos: An information aesthetic for comparative genomics. Genome Research, 19, 1639–1645. 19541911 10.1101/gr.092759.109 PMC2752132

[mbo370000-bib-0085] Kumar, J. , Singh, D. , Tyagi, M. B. , & Kumar, A. (2019). Cyanobacteria: Applications in biotechnology. In Cyanobacteria From Basic Science to Applications (pp. 327–346).

[mbo370000-bib-0086] Kumar, S. , Stecher, G. , Li, M. , Knyaz, C. , & Tamura, K. (2018). MEGA X: Molecular evolutionary genetics analysis across computing platforms. Molecular Biology and Evolution, 35(6), 1547–1549. 29722887 10.1093/molbev/msy096 PMC5967553

[mbo370000-bib-0087] Kunin, V. , He, S. , Warnecke, F. , Peterson, S. B. , Garcia Martin, H. , Haynes, M. , Ivanova, N. , Blackall, L. L. , Breitbart, M. , Rohwer, F. , McMahon, K. D. , & Hugenholtz, P. (2008). A bacterial metapopulation adapts locally to phage predation despite global dispersal. Genome Research, 18(2), 293–297. 18077539 10.1101/gr.6835308 PMC2203627

[mbo370000-bib-0088] Lacap, D. C. , Barraquio, W. , & Pointing, S. B. (2007). Thermophilic microbial mats in a tropical geothermal location display pronounced seasonal changes but appear resilient to stochastic disturbance. Environmental Microbiology, 9(12), 3065–3076. 17991034 10.1111/j.1462-2920.2007.01417.x

[mbo370000-bib-0089] Lang, M. , Krin, E. , Korlowski, C. , Sismeiro, O. , Varet, H. , Coppée, J. Y. , Mazel, D. , & Baharoglu, Z. (2021). Sleeping ribosomes: Bacterial signaling triggers RaiA‐mediated persistence to aminoglycosides. iScience, 24(10), 103128. 34611612 10.1016/j.isci.2021.103128 PMC8476650

[mbo370000-bib-0090] Lau, M. C. Y. , Aitchison, J. C. , & Pointing, S. B. (2009). Bacterial community composition in thermophilic microbial mats from five hot springs in central Tibet. Extremophiles, 13(1), 139–149. 19023516 10.1007/s00792-008-0205-3

[mbo370000-bib-0091] Letunic, I. , & Bork, P. (2024). Interactive Tree of Life (iTOL) v6: Recent updates to the phylogenetic tree display and annotation tool. Nucleic Acids Research, 52, gkae268. 10.1093/nar/gkae268 PMC11223838 38613393

[mbo370000-bib-0092] Leu, J.‐Y. , Lin, T.‐H. , Selvamani, M. J. P. , Chen, H.‐C. , Liang, J.‐Z. , & Pan, K.‐M. (2013). Characterization of a novel thermophilic cyanobacterial strain from Taian hot springs in Taiwan for high CO_2_ mitigation and C‐phycocyanin extraction. Process Biochemistry, 48(1), 41–48.

[mbo370000-bib-0093] Li, W. , O'Neill, K. R. , Haft, D. H. , DiCuccio, M. , Chetvernin, V. , Badretdin, A. , Coulouris, G. , Chitsaz, F. , Derbyshire, M. K. , Durkin, A. S. , Gonzales, N. R. , Gwadz, M. , Lanczycki, C. J. , Song, J. S. , Thanki, N. , Wang, J. , Yamashita, R. A. , Yang, M. , Zheng, C. , … Thibaud‐Nissen, F. (2021). RefSeq: Expanding the prokaryotic genome annotation pipeline reach with protein family model curation. Nucleic Acids Research, 49(D1), D1020–D1028. 33270901 10.1093/nar/gkaa1105 PMC7779008

[mbo370000-bib-0094] Lobry, J. R. (1996). Asymmetric substitution patterns in the two DNA strands of bacteria. Molecular Biology and Evolution, 13(5), 660–665. 8676740 10.1093/oxfordjournals.molbev.a025626

[mbo370000-bib-0095] Loftie‐Eaton, W. , Taylor, M. , Horne, K. , Tuffin, M. I. , Burton, S. G. , & Cowan, D. A. (2013). Balancing redox cofactor generation and ATP synthesis: Key microaerobic responses in thermophilic fermentations. Biotechnology and Bioengineering, 110(4), 1057–1065. 23124997 10.1002/bit.24774

[mbo370000-bib-0096] Lomsadze, A. , Gemayel, K. , Tang, S. , & Borodovsky, M. (2018). Modeling leaderless transcription and atypical genes results in more accurate gene prediction in prokaryotes. Genome Research, 28(7), 1079–1089. 29773659 10.1101/gr.230615.117 PMC6028130

[mbo370000-bib-0097] Low, D. A. , Weyand, N. J. , & Mahan, M. J. (2001). Roles of DNA adenine methylation in regulating bacterial gene expression and virulence. Infection and Immunity, 69(12), 7197–7204. 11705888 10.1128/IAI.69.12.7197-7204.2001 PMC98802

[mbo370000-bib-0098] Lowe, T. M. , & Eddy, S. R. (1997). tRNAscan‐SE: A program for improved detection of transfer RNA genes in genomic sequence. Nucleic Acids Research, 25(5), 955–964. 9023104 10.1093/nar/25.5.955 PMC146525

[mbo370000-bib-0099] Ma, B. , Zhang, K. , Hendrie, C. , Liang, C. , Li, M. , Doherty‐Kirby, A. , & Lajoie, G. (2003). PEAKS: Powerful software for peptide de novo sequencing by tandem mass spectrometry. Rapid Communications in Mass Spectrometry, 17(20), 2337–2342. 14558135 10.1002/rcm.1196

[mbo370000-bib-0100] Macinga, D. R. , Cook, G. M. , Poole, R. K. , & Rather, P. N. (1998). Identification and characterization of aarF, a locus required for production of ubiquinone in *Providencia stuartii* and *Escherichia coli* and for expression of 2′‐N‐acetyltransferase in *P. stuartii* . Journal of Bacteriology, 180(1), 128–135. 9422602 10.1128/jb.180.1.128-135.1998 PMC106858

[mbo370000-bib-0101] Magnuson, R. D. (2007). Hypothetical functions of toxin‐antitoxin systems. Journal of Bacteriology, 189(17), 6089–6092. 17616596 10.1128/JB.00958-07 PMC1951896

[mbo370000-bib-0102] Maguire, B. A. , & Wild, D. G. (1997). The roles of proteins L28 and L33 in the assembly and function of *Escherichia coli* ribosomes in vivo. Molecular Microbiology, 23(2), 237–245. 9044258 10.1046/j.1365-2958.1997.2131578.x

[mbo370000-bib-0103] Manni, M. , Berkeley, M. R. , Seppey, M. , Simão, F. A. , & Zdobnov, E. M. (2021). BUSCO Update: novel and streamlined workflows along with broader and deeper phylogenetic coverage for scoring of eukaryotic, prokaryotic, and viral genomes. Molecular Biology and Evolution, 38(10), 4647–4654. 34320186 10.1093/molbev/msab199 PMC8476166

[mbo370000-bib-0104] Mapelli‐Brahm, P. , & Meléndez‐Martínez, A. J. (2021). The colourless carotenoids phytoene and phytofluene: sources, consumption, bioavailability and health effects. Current Opinion in Food Science, 41, 201–209.

[mbo370000-bib-0105] Mascarenhas, J. (2002). Cell cycle‐dependent localization of two novel prokaryotic chromosome segregation and condensation proteins in *Bacillus subtilis* that interact with SMC protein. The EMBO Journal, 21(12), 3108–3118. 12065423 10.1093/emboj/cdf314 PMC126067

[mbo370000-bib-0106] Maslova, I. P. , Mouradyan, E. A. , Lapina, S. S. , Klyachko‐Gurvich, G. L. , & Los, D. A. (2004). Lipid fatty acid composition and thermophilicity of Cyanobacteria. Russian Journal of Plant Physiology, 51(3), 353–360.

[mbo370000-bib-0107] Mathews‐Roth, M. M. (1982). Antitumor activity of β‐Carotene, canthaxanthin and phytoene. Oncology, 39(1), 33–37. 6799883 10.1159/000225601

[mbo370000-bib-0108] McLennan, A. G. (2006). The Nudix hydrolase superfamily. Cellular and Molecular Life Sciences, 63(2), 123–143. 16378245 10.1007/s00018-005-5386-7 PMC11136074

[mbo370000-bib-0109] Meier‐Kolthoff, J. P. , Klenk, H.‐P. , & Göker, M. (2014). Taxonomic use of DNA G+C content and DNA–DNA hybridization in the genomic age. International Journal of Systematic and Evolutionary Microbiology, 64(Pt_2), 352–356. 24505073 10.1099/ijs.0.056994-0

[mbo370000-bib-0110] Meier‐Kolthoff, J. P. , Auch, A. F. , Klenk, H.‐P. , & Göker, M. (2013). Genome sequence‐based species delimitation with confidence intervals and improved distance functions. BMC Bioinformatics, 14(1), 60. 23432962 10.1186/1471-2105-14-60 PMC3665452

[mbo370000-bib-0111] Meier‐Kolthoff, J. P. , Carbasse, J. S. , Peinado‐Olarte, R. L. , & Göker, M. (2022). TYGS and LPSN: A database tandem for fast and reliable genome‐based classification and nomenclature of prokaryotes. Nucleic Acids Research, 50(D1), D801–D807. 34634793 10.1093/nar/gkab902 PMC8728197

[mbo370000-bib-0112] Meléndez‐Martínez, A. J. , Paulino, M. , Stinco, C. M. , Mapelli‐Brahm, P. , & Wang, X. D. (2014). Study of the time‐course of cis/trans (Z/E) isomerization of lycopene, phytoene, and phytofluene from tomato. Journal of Agricultural and Food Chemistry, 62(51), 12399–12406. 25426993 10.1021/jf5041965

[mbo370000-bib-0113] Miller, S. R. , & Carvey, D. (2019). Ecological divergence with gene flow in a thermophilic cyanobacterium. Microbial Ecology, 78(1), 33–41. 30267129 10.1007/s00248-018-1267-0

[mbo370000-bib-0114] Miyatake, K. , Nakano, Y. , & Kitaoka, S. (1979). [41] Pantothenate synthetase from *Escherichia coli* [d‐pantoate: β‐alanine ligase (AMP‐forming), EC 6.3.2.1]. Methods in Enzymology, 62(C), 215–219. 374975 10.1016/0076-6879(79)62221-8

[mbo370000-bib-0115] Mullen, C. A. , & Jennings, P. A. (1996). Glycinamide ribonucleotide transformylase undergoes pH‐dependent dimerization. Journal of Molecular Biology, 262(5), 746–755. 8876651 10.1006/jmbi.1996.0549

[mbo370000-bib-0116] Müller, J. E. N. , Litsanov, B. , Bortfeld‐Miller, M. , Trachsel, C. , Grossmann, J. , Brautaset, T. , & Vorholt, J. A. (2014). Proteomic analysis of the thermophilic methylotroph *Bacillus methanolicus* MGA3. Proteomics, 14(6), 725–737. 24452867 10.1002/pmic.201300515

[mbo370000-bib-0117] Nei, M. , & Kumar, S. (2000). Molecular evolution and phylogenetics. Oxford University Press.

[mbo370000-bib-0118] Nelson, K. J. , Knutson, S. T. , Soito, L. , Klomsiri, C. , Poole, L. B. , & Fetrow, J. S. (2011). Analysis of the peroxiredoxin family: using active site structure and sequence information for global classification and residue analysis. Proteins: Structure, Function, and Bioinformatics, 79(3), 947–964. 10.1002/prot.22936 PMC3065352 21287625

[mbo370000-bib-0119] Nishioka, M. , Nakai, K. , Miyake, M. , Asada, Y. , & Taya, M. (2001). Production of poly‐β‐hydroxybutyrate by thermophilic cyanobacterium, *Synechococcus* sp. MA19, under phosphate‐limited conditions. Biotechnology Letters, 23(14), 1095–1099.

[mbo370000-bib-0120] Oren, A. (2015). Cyanobacteria in hypersaline environments: Biodiversity and physiological properties. Biodiversity and Conservation, 24(4), 781–798.

[mbo370000-bib-0121] Overbeek, R. , Olson, R. , Pusch, G. D. , Olsen, G. J. , Davis, J. J. , Disz, T. , Edwards, R. A. , Gerdes, S. , Parrello, B. , Shukla, M. , Vonstein, V. , Wattam, A. R. , Xia, F. , & Stevens, R. (2014). The SEED and the rapid annotation of microbial genomes using subsystems technology (RAST). Nucleic Acids Research, 42(D1), D206–D214. 24293654 10.1093/nar/gkt1226 PMC3965101

[mbo370000-bib-0122] Palmer, K. L. , & Gilmore, M. S. (2010). Multidrug‐resistant enterococci lack CRISPR‐cas. mBio, 1(4), 227–237. 10.1128/mBio.00227-10 PMC2975353 21060735

[mbo370000-bib-0123] Pandey, D. P. (2005). Toxin‐antitoxin loci are highly abundant in free‐living but lost from host‐associated prokaryotes. Nucleic Acids Research, 33(3), 966–976. 15718296 10.1093/nar/gki201 PMC549392

[mbo370000-bib-0124] Paper, M. , Koch, M. , Jung, P. , Lakatos, M. , Nilges, T. , & Brück, T. B. (2023). Rare earths stick to rare Cyanobacteria: Future potential for bioremediation and recovery of rare earth elements. Frontiers in Bioengineering and Biotechnology, 11, 1–14. 10.3389/fbioe.2023.1130939 PMC10011134 36926689

[mbo370000-bib-0125] Papke, R. T. , Ramsing, N. B. , Bateson, M. M. , & Ward, D. M. (2003). Geographical isolation in hot spring Cyanobacteria. Environmental Microbiology, 5(8), 650–659. 12871232 10.1046/j.1462-2920.2003.00460.x

[mbo370000-bib-0126] Parks, D. H. , Imelfort, M. , Skennerton, C. T. , Hugenholtz, P. , & Tyson, G. W. (2015). CheckM: Assessing the quality of microbial genomes recovered from isolates, single cells, and metagenomes. Genome Research, 25, 1043–1055. 25977477 10.1101/gr.186072.114 PMC4484387

[mbo370000-bib-0127] Patel, A. , Matsakas, L. , Rova, U. , & Christakopoulos, P. (2019). A perspective on biotechnological applications of thermophilic microalgae and Cyanobacteria. Bioresource Technology, 278, 424–434. 30685131 10.1016/j.biortech.2019.01.063

[mbo370000-bib-0128] Paul, R. , Weiser, S. , Amiot, N. C. , Chan, C. , Schirmer, T. , Giese, B. , & Jenal, U. (2004). Cell cycle‐dependent dynamic localization of a bacterial response regulator with a novel di‐guanylate cyclase output domain. Genes & Development, 18(6), 715–727. 15075296 10.1101/gad.289504 PMC387245

[mbo370000-bib-0129] Paysan‐Lafosse, T. , Blum, M. , Chuguransky, S. , Grego, T. , Pinto, B. L. , Salazar, G. A. , Bileschi, M. L. , Bork, P. , Bridge, A. , Colwell, L. , Gough, J. , Haft, D. H. , Letunić, I. , Marchler‐Bauer, A. , Mi, H. , Natale, D. A. , Orengo, C. A. , Pandurangan, A. P. , Rivoire, C. , … Bateman, A. (2023). InterPro in 2022. Nucleic Acids Research, 51(D1), D418–D427. 36350672 10.1093/nar/gkac993 PMC9825450

[mbo370000-bib-0130] Paz, A. , Mester, D. , Baca, I. , Nevo, E. , & Korol, A. (2004). Adaptive role of increased frequency of polypurine tracts in mRNA sequences of thermophilic prokaryotes. Proceedings of the National Academy of Sciences, 101(9), 2951–2956. 10.1073/pnas.0308594100 PMC365726 14973185

[mbo370000-bib-0800] Polack, F. P. , Thomas, S. J. , Kitchin, N. , Absalon, J. , Gurtman, A. , Lockhart, S. , Perez, J. L. , Pérez Marc, G. , Moreira, E. D. , Zerbini, C. , Bailey, R. , Swanson, K. A. , Roychoudhury, S. , Koury, K. , Li, P. , Kalina, W. V. , Cooper, D. , Frenck, R. W. Jr. , Hammitt, L. L. , … Gruber, W. C. (2020). Safety and efficacy of the BNT162b2 mRNA Covid‐19 vaccine. The New England Journal of Medicine, 383(27), 2603–2615. 33301246 10.1056/NEJMoa2034577 PMC7745181

[mbo370000-bib-0131] Poon, W. W. , Davis, D. E. , Ha, H. T. , Jonassen, T. , Rather, P. N. , & Clarke, C. F. (2000). Identification of *Escherichia coli* ubiB, a gene required for the first monooxygenase step in ubiquinone biosynthesis. Journal of Bacteriology, 182(18), 5139–5146. 10960098 10.1128/jb.182.18.5139-5146.2000 PMC94662

[mbo370000-bib-0132] Portillo, M. C. , Sririn, V. , Kanoksilapatham, W. , & Gonzalez, J. M. (2009). Differential microbial communities in hot spring mats from Western Thailand. Extremophiles, 13(2), 321–331. 19109691 10.1007/s00792-008-0219-x

[mbo370000-bib-0133] Pysz, M. A. , Shockley, K. R. , Montero, C. I. , Conners, S. B. , Johnson, M. R. , Kelly, R. M. , & Ward, D. E. (2004). Transcriptional analysis of dynamic heat‐shock response by the hyperthermophilic bacterium *Thermotoga maritima* . Extremophiles, 8(3), 209–217. 14991425 10.1007/s00792-004-0379-2

[mbo370000-bib-0134] Rai, R. , Singh, S. , Rai, K. K. , Raj, A. , Sriwastaw, S. , & Rai, L. C. (2021). Regulation of antioxidant defense and glyoxalase systems in Cyanobacteria. Plant Physiology and Biochemistry, 168, 353–372. 34700048 10.1016/j.plaphy.2021.09.037

[mbo370000-bib-0135] Razew, A. , Schwarz, J. N. , Mitkowski, P. , Sabala, I. , & Kaus‐Drobek, M. (2022). One fold, many functions—M23 family of peptidoglycan hydrolases. Frontiers in Microbiology, 13, 1036964. 36386627 10.3389/fmicb.2022.1036964 PMC9662197

[mbo370000-bib-0136] Reeves, S. A. , Parsonage, D. , Nelson, K. J. , & Poole, L. B. (2011). Kinetic and thermodynamic features reveal that *Escherichia coli* BCP is an unusually versatile peroxiredoxin. Biochemistry, 50(41), 8970–8981. 21910476 10.1021/bi200935d PMC3204386

[mbo370000-bib-0137] Ritz, M. , Ahmad, N. , Brueck, T. , & Mehlmer, N. (2023). Comparative genome‐wide analysis of two caryopteris x clandonensis cultivars: insights on the biosynthesis of volatile terpenoids. Plants, 12(3), 632. 36771729 10.3390/plants12030632 PMC9921992

[mbo370000-bib-0138] Ronquist, F. , & Huelsenbeck, J. P. (2003). MrBayes 3: Bayesian phylogenetic inference under mixed models. Bioinformatics, 19(12), 1572–1574. 12912839 10.1093/bioinformatics/btg180

[mbo370000-bib-0139] Ryjenkov, D. A. , Tarutina, M. , Moskvin, O. V. , & Gomelsky, M. (2005). Cyclic diguanylate is a ubiquitous signaling molecule in bacteria: Insights into biochemistry of the GGDEF protein domain. Journal of Bacteriology, 187(5), 1792–1798. 15716451 10.1128/JB.187.5.1792-1798.2005 PMC1064016

[mbo370000-bib-0140] Sabath, N. , Ferrada, E. , Barve, A. , & Wagner, A. (2013). Growth temperature and genome size in bacteria are negatively correlated, suggesting genomic streamlining during thermal adaptation. Genome Biology and Evolution, 5(5), 966–977. 23563968 10.1093/gbe/evt050 PMC3673621

[mbo370000-bib-0141] Sato, T. , Kouda, M. , & Hoshino, T. (2004). Site‐directed mutagenesis experiments on the putative deprotonation site of squalene‐hopene cyclase from *Alicyclobacillus acidocaldarius* . Bioscience, Biotechnology, and Biochemistry, 68(3), 728–738. 15056909 10.1271/bbb.68.728

[mbo370000-bib-0142] Sato, T. , Hoshino, H. , Yoshida, S. , Nakajima, M. , & Hoshino, T. (2011a). Bifunctional triterpene/sesquarterpene cyclase: Tetraprenyl‐β‐ curcumene cyclase is also squalene cyclase in *Bacillus megaterium* . Journal of the American Chemical Society, 133(44), 17540–17543. 21981578 10.1021/ja2060319

[mbo370000-bib-0143] Sato, T. , Yoshida, S. , Hoshino, H. , Tanno, M. , Nakajima, M. , & Hoshino, T. (2011b). Sesquarterpenes (C35 terpenes) biosynthesized via the cyclization of a linear C35 isoprenoid by a tetraprenyl‐β‐ curcumene synthase and a tetraprenyl‐β‐curcumene cyclase: Identification of a new terpene cyclase. Journal of the American Chemical Society, 133(25), 9734–9737. 21627333 10.1021/ja203779h

[mbo370000-bib-0144] Sawayama, S. , Rao, K. K. , & Hall, D. O. (1998). Nitrate and phosphate ion removal from water by *Phormidium laminosum* immobilized on hollow fibres in a photobioreactor. Applied Microbiology and Biotechnology, 49(4), 463–468.

[mbo370000-bib-0145] Saxena, R. , Dhakan, D. B. , Mittal, P. , Waiker, P. , Chowdhury, A. , Ghatak, A. , & Sharma, V. K. (2017). Metagenomic analysis of hot springs in Central India reveals hydrocarbon degrading thermophiles and pathways essential for survival in extreme environments. Frontiers in Microbiology, 7, 2123. 28105025 10.3389/fmicb.2016.02123 PMC5214690

[mbo370000-bib-0146] Schmutzer, M. , & Barraclough, T. G. (2019). The role of recombination, niche‐specific gene pools and flexible genomes in the ecological speciation of bacteria. Ecology and Evolution, 9(8), 4544–4556. 31031926 10.1002/ece3.5052 PMC6476844

[mbo370000-bib-0147] Seemann, T. (2013). *Barrnap: Rapid ribosomal RNA prediction*. Software from github. https://github.com/tseemann/barrnap

[mbo370000-bib-0148] Selengut, J. D. , Haft, D. H. , Davidsen, T. , Ganapathy, A. , Gwinn‐Giglio, M. , Nelson, W. C. , Richter, A. R. , & White, O. (2007). TIGRFAMs and genome properties: Tools for the assignment of molecular function and biological process in prokaryotic genomes. Nucleic Acids Research, 35(Database issue), D260–D264. 17151080 10.1093/nar/gkl1043 PMC1781115

[mbo370000-bib-0149] Shcherbakov, D. , Dontsova, M. , Tribus, M. , Garber, M. , & Piendl, W. (2006). Stability of the ‘L12 stalk’ in ribosomes from mesophilic and (hyper)thermophilic archaea and bacteria. Nucleic Acids Research, 34(20), 5800–5814. 17053098 10.1093/nar/gkl751 PMC1635324

[mbo370000-bib-0150] Shih, P. M. , Wu, D. , Latifi, A. , Axen, S. D. , Fewer, D. P. , Talla, E. , Calteau, A. , Cai, F. , Tandeau de Marsac, N. , Rippka, R. , Herdman, M. , Sivonen, K. , Coursin, T. , Laurent, T. , Goodwin, L. , Nolan, M. , Davenport, K. W. , Han, C. S. , Rubin, E. M. , … Kerfeld, C. A. (2013). Improving the coverage of the cyanobacterial phylum using diversity‐driven genome sequencing. Proceedings of the National Academy of Sciences, 110(3), 1053–1058. 10.1073/pnas.1217107110 PMC3549136 23277585

[mbo370000-bib-0151] Shih, T. W. , & Pan, T. M. (2011). Stress responses of thermophilic *Geobacillus* sp. NTU 03 caused by heat and heat‐induced stress. Microbiological Research, 166(5), 346–359. 20869219 10.1016/j.micres.2010.08.001

[mbo370000-bib-0152] Shirmast, P. , Ghafoori, S. M. , Irwin, R. M. , Abendroth, J. , Mayclin, S. J. , Lorimer, D. D. , Edwards, T. E. , & Forwood, J. K. (2021). Structural characterization of a GNAT family acetyltransferase from *Elizabethkingia anophelis* bound to acetyl‐CoA reveals a new dimeric interface. Scientific Reports, 11(1), 1–9. 33446675 10.1038/s41598-020-79649-5 PMC7809356

[mbo370000-bib-0153] Shmakov, S. , Abudayyeh, O. O. , Makarova, K. S. , Wolf, Y. I. , Gootenberg, J. S. , Semenova, E. , Minakhin, L. , Joung, J. , Konermann, S. , Severinov, K. , Zhang, F. , & Koonin, E. V. (2015). Discovery and functional characterization of diverse class 2 CRISPR‐Cas systems. Molecular Cell, 60(3), 385–397. 26593719 10.1016/j.molcel.2015.10.008 PMC4660269

[mbo370000-bib-0154] Shockley, K. R. , Ward, D. E. , Chhabra, S. R. , Conners, S. B. , Montero, C. I. , & Kelly, R. M. (2003). Heat shock response by the hyperthermophilic archaeon *Pyrococcus furiosus* . Applied and Environmental Microbiology, 69(4), 2365–2371. 12676722 10.1128/AEM.69.4.2365-2371.2003 PMC154833

[mbo370000-bib-0155] Singh, R. K. , Tiwari, S. P. , Rai, A. K. , & Mohapatra, T. M. (2011). Cyanobacteria: An emerging source for drug discovery. The Journal of Antibiotics, 64(6), 401–412. 21468079 10.1038/ja.2011.21

[mbo370000-bib-0156] Sompong, U. , Anuntalabhochai, S. , W. Cutler, R. , W. Castenholz, R. , & Peerapornpisal, Y. (2008). Morphological and phylogenic diversity of cyanobacterial populations in six hot springs of Thailand. ScienceAsia, 34(2), 153–162.

[mbo370000-bib-0157] Soppa, J. , Kobayashi, K. , Noirot‐Gros, M. F. , Oesterhelt, D. , Ehrlich, S. D. , Dervyn, E. , Ogasawara, N. , & Moriya, S. (2002). Discovery of two novel families of proteins that are proposed to interact with prokaryotic SMC proteins, and characterization of the *Bacillus subtilis* family members ScpA and ScpB. Molecular Microbiology, 45(1), 59–71. 12100548 10.1046/j.1365-2958.2002.03012.x

[mbo370000-bib-0158] Stanier, R. Y. , Kunisawa, R. , Mandel, M. , & Cohen‐Bazire, G. (1971). Purification and properties of unicellular blue‐green algae (order Chroococcales). Bacteriological Reviews, 35(2), 171–205. 4998365 10.1128/br.35.2.171-205.1971 PMC378380

[mbo370000-bib-0159] Stolyar, S. , Liu, Z. , Thiel, V. , Tomsho, L. P. , Pinel, N. , Nelson, W. C. , Lindemann, S. R. , Romine, M. F. , Haruta, S. , Schuster, S. C. , Bryant, D. A. , & Fredrickson, J. K. (2014). Genome sequence of the thermophilic cyanobacterium *Thermosynechococcus* sp. strain NK55a. Genome Announcements, 2(1), 10–1128. 10.1128/genomeA.01060-13 PMC3907722 24482507

[mbo370000-bib-0160] Strunecký, O. , Ivanova, A. P. , & Mareš, J. (2023). An updated classification of cyanobacterial orders and families based on phylogenomic and polyphasic analysis. Journal of Phycology, 59(1), 12–51. 36443823 10.1111/jpy.13304

[mbo370000-bib-0161] Studer, G. , Rempfer, C. , Waterhouse, A. M. , Gumienny, R. , Haas, J. , & Schwede, T. (2020). QMEANDisCo‐distance constraints applied on model quality estimation. Bioinformatics, 36(6), 1765–1771. 31697312 10.1093/bioinformatics/btz828 PMC7075525

[mbo370000-bib-0162] Suttisansanee, U. , & Honek, J. F. (2011). Bacterial glyoxalase enzymes. Seminars in Cell & Developmental Biology, 22(3), 285–292. 21310258 10.1016/j.semcdb.2011.02.004

[mbo370000-bib-0163] Suzuki, E. , Onoda, M. , Colleoni, C. , Ball, S. , Fujita, N. , & Nakamura, Y. (2013). Physicochemical variation of cyanobacterial starch, the insoluble α‐glucans in Cyanobacteria. Plant and Cell Physiology, 54(4), 465–473. 23299410 10.1093/pcp/pcs190

[mbo370000-bib-0164] Tang, J. , Jiang, Y. , Hu, Z. , Zhou, H. , You, D. , & Daroch, M. (2024). Genomic and phenotypic characterization of Thermosynechococcus‐like strains reveals eight species within the genus Thermosynechococcus and a novel genus *Parathermosynechococcus* gen. nov. Molecular Phylogenetics and Evolution, 197, 108094. 38723792 10.1016/j.ympev.2024.108094

[mbo370000-bib-0165] Tang, J. , Jiang, D. , Luo, Y. , Liang, Y. , Li, L. , Shah, M. M. R. , & Daroch, M. (2018). Potential new genera of cyanobacterial strains isolated from thermal springs of Western Sichuan, China. Algal Research, 31, 14–20.

[mbo370000-bib-0166] Tang, J. , Zhou, H. , Yao, D. , Riaz, S. , You, D. , Klepacz‐Smółka, A. , & Daroch, M. (2022). Comparative genomic analysis revealed distinct molecular components and organization of CO_2_‐concentrating mechanism in thermophilic Cyanobacteria. Frontiers in Microbiology, 13, 1–17. 10.3389/fmicb.2022.876272 PMC9120777 35602029

[mbo370000-bib-0167] Tatusova, T. , DiCuccio, M. , Badretdin, A. , Chetvernin, V. , Nawrocki, E. P. , Zaslavsky, L. , Lomsadze, A. , Pruitt, K. D. , Borodovsky, M. , & Ostell, J. (2016). NCBI prokaryotic genome annotation pipeline. Nucleic Acids Research, 44(14), 6614–6624. 27342282 10.1093/nar/gkw569 PMC5001611

[mbo370000-bib-0168] Team, P. (2022). RStudio: Integrated development environment for R. Posit Software, PBC.

[mbo370000-bib-0169] Thompson, M. J. , & Eisenberg, D. (1999). Transproteomic evidence of a loop‐deletion mechanism for enhancing protein thermostability. Journal of Molecular Biology, 290(2), 595–604. 10390356 10.1006/jmbi.1999.2889

[mbo370000-bib-0170] Trauger, S. A. , Kalisak, E. , Kalisiak, J. , Morita, H. , Weinberg, M. V. , Menon, A. L. , Poole II, F. L. , Adams, M. W. W. , & Siuzdak, G. (2008). Correlating the transcriptome, proteome, and metabolome in the environmental adaptation of a hyperthermophile. Journal of Proteome Research, 7(3), 1027–1035. 18247545 10.1021/pr700609j

[mbo370000-bib-0171] Tsilibaris, V. , Maenhaut‐Michel, G. , Mine, N. , & Van Melderen, L. (2007). What is the benefit to *Escherichia coli* of having multiple toxin‐antitoxin systems in its genome? Journal of Bacteriology, 189(17), 6101–6108. 17513477 10.1128/JB.00527-07 PMC1951899

[mbo370000-bib-0172] Ueta, M. , Yoshida, H. , Wada, C. , Baba, T. , Mori, H. , & Wada, A. (2005). Ribosome binding proteins YhbH and YfiA have opposite functions during 100S formation in the stationary phase of *Escherichia coli* . Genes to Cells, 10(12), 1103–1112. 16324148 10.1111/j.1365-2443.2005.00903.x

[mbo370000-bib-0173] Van Noort, V. , Bradatsch, B. , Arumugam, M. , Amlacher, S. , Bange, G. , Creevey, C. , Falk, S. , Mende, D. R. , Sinning, I. , Hurt, E. , & Bork, P. (2013). Consistent mutational paths predict eukaryotic thermostability. BMC Evolutionary Biology, 13(1), 7. 23305080 10.1186/1471-2148-13-7 PMC3546890

[mbo370000-bib-0174] Verkhovsky, M. I. , & Bogachev, A. V. (2010). Sodium‐translocating NADH:quinone oxidoreductase as a redox‐driven ion pump. Biochimica et Biophysica Acta (BBA) ‐ Bioenergetics, 1797(6–7), 738–746. 20056102 10.1016/j.bbabio.2009.12.020

[mbo370000-bib-0175] Vobruba, S. , Kadlcik, S. , Janata, J. , & Kamenik, Z. (2022). TldD/TldE peptidases and N‐deacetylases: A structurally unique yet ubiquitous protein family in the microbial metabolism. Microbiological Research, 265, 127186. 36155963 10.1016/j.micres.2022.127186

[mbo370000-bib-0176] Wang, J. , Zhao, C. , Meng, B. , Xie, J. , Zhou, C. , Chen, X. , Zhao, K. , Shao, J. , Xue, Y. , Xu, N. , Ma, Y. , & Liu, S. (2007). The proteomic alterations of *Thermoanaerobacter tengcongensis* cultured at different temperatures. Proteomics, 7(9), 1409–1419. 17469076 10.1002/pmic.200500226

[mbo370000-bib-0177] Wang, Q. , Cen, Z. , & Zhao, J. (2015). The survival mechanisms of thermophiles at high temperatures: An angle of omics. Physiology, 30(2), 97–106. 25729055 10.1152/physiol.00066.2013

[mbo370000-bib-0178] Wang, Z. , Tong, W. , Wang, Q. , Bai, X. , Chen, Z. , Zhao, J. , Xu, N. , & Liu, S. (2012). The temperature dependent proteomic analysis of *Thermotoga maritima* . PLoS One, 7(10), e46463. 23071576 10.1371/journal.pone.0046463 PMC3465335

[mbo370000-bib-0179] Wang, Z. , Pan, Q. , Gendron, P. , Zhu, W. , Guo, F. , Cen, S. , Wainberg, M. A. , & Liang, C. (2016). CRISPR/Cas9‐derived mutations both inhibit HIV‐1 replication and accelerate viral escape. Cell Reports, 15(3), 481–489. 27068471 10.1016/j.celrep.2016.03.042

[mbo370000-bib-0180] Ward, D. M. , Castenholz, R. W. , & Miller, S. R. (2012). Cyanobacteria in Geothermal Habitats. In B. A. Whitton (Ed.), Ecology of Cyanobacteria II: Their Diversity in Space and Time. Springer.

[mbo370000-bib-0181] Ward, L. M. , Idei, A. , Terajima, S. , Kakegawa, T. , Fischer, W. W. , & McGlynn, S. E. (2017). Microbial diversity and iron oxidation at Okuoku‐hachikurou Onsen, a Japanese hot spring analog of Precambrian iron formations. Geobiology, 15(6), 817–835. 29035022 10.1111/gbi.12266

[mbo370000-bib-0182] Watanabe, F. , Katsura, H. , Takenaka, S. , Fujita, T. , Abe, K. , Tamura, Y. , Nakatsuka, T. , & Nakano, Y. (1999). Pseudovitamin B12 is the predominant cobamide of an algal health food, spirulina tablets. Journal of Agricultural and Food Chemistry, 47(11), 4736–4741. 10552882 10.1021/jf990541b

[mbo370000-bib-0183] Waterhouse, A. , Bertoni, M. , Bienert, S. , Studer, G. , Tauriello, G. , Gumienny, R. , Heer, F. T. , de Beer, T. A. P. , Rempfer, C. , Bordoli, L. , Lepore, R. , & Schwede, T. (2018). SWISS‐MODEL: Homology modelling of protein structures and complexes. Nucleic Acids Research, 46(W1), W296–W303. 29788355 10.1093/nar/gky427 PMC6030848

[mbo370000-bib-0184] Weissbach, H. , Etienne, F. , Hoshi, T. , Heinemann, S. H. , Lowther, W. T. , Matthews, B. , St. John, G. , Nathan, C. , & Brot, N. (2002). Peptide methionine sulfoxide reductase: Structure, mechanism of action, and biological function. Archives of Biochemistry and Biophysics, 397(2), 172–178. 11795868 10.1006/abbi.2001.2664

[mbo370000-bib-0185] Westra, E. R. , Dowling, A. J. , Broniewski, J. M. , & van Houte, S. (2016). Evolution and ecology of CRISPR. Annual Review of Ecology, Evolution, and Systematics, 47(1), 307–331.

[mbo370000-bib-0186] Wickham, H. (2008). Elegant graphics for data analysis: ggplot2. In Applied Spatial Data Analysis with R (pp. 1–54).

[mbo370000-bib-0187] Wyatt, M. D. , Allan, J. M. , Lau, A. Y. , Ellenberger, T. E. , & Samson, L. D. (1999). 3‐methyladenine DNA glycosylases: structure, function, and biological importance. BioEssays, 21(8), 668–676. 10440863 10.1002/(SICI)1521-1878(199908)21:8<668::AID-BIES6>3.0.CO;2-D

[mbo370000-bib-0188] Yeats, C. , Bentley, S. , & Bateman, A. (2003). New knowledge from old: In silico discovery of novel protein domains in *Streptomyces coelicolor* . BMC Microbiology, 3(1), 3. 12625841 10.1186/1471-2180-3-3 PMC151604

[mbo370000-bib-0189] Yoon, S. H. , Ha, S. , Lim, J. , Kwon, S. , & Chun, J. (2017). A large‐scale evaluation of algorithms to calculate average nucleotide identity. Antonie Van Leeuwenhoek, 110(10), 1281–1286. 28204908 10.1007/s10482-017-0844-4

[mbo370000-bib-0190] Yoshihara, S. , & Ikeuchi, M. (2004). Phototactic motility in the unicellular cyanobacterium *Synechocystis* sp. PCC 6803. Photochemical & Photobiological Sciences, 3(6), 512–518. 15170479 10.1039/b402320j

[mbo370000-bib-0191] Zeldovich, K. B. , Berezovsky, I. N. , & Shakhnovich, E. I. (2007). Protein and DNA sequence determinants of thermophilic adaptation. PLoS Computational Biology, 3(1), e5. 17222055 10.1371/journal.pcbi.0030005 PMC1769408

[mbo370000-bib-0192] Zhang, P. , Zhang, Z. , Zhang, L. , Wang, J. , & Wu, C. (2020). Glycosyltransferase GT1 family: Phylogenetic distribution, substrates coverage, and representative structural features. Computational and Structural Biotechnology Journal, 18, 1383–1390. 32637037 10.1016/j.csbj.2020.06.003 PMC7316871

[mbo370000-bib-0193] Zhang, Z. , Schwartz, S. , Wagner, L. , & Miller, W. (2000). A greedy algorithm for aligning DNA sequences. Journal of Computational Biology, 7(1–2), 203–214. 10890397 10.1089/10665270050081478

[mbo370000-bib-0194] Zuo, Y. , Wang, Y. , & Malhotra, A. (2005). Crystal structure of *Escherichia coli* RNase D, an exoribonuclease involved in structured RNA processing. Structure, 13(7), 973–984. 16004870 10.1016/j.str.2005.04.015

